# Intracellular Measurement-Informed Multiscale Modeling for Scalable iPSC Manufacturing

**Published:** 2026-03-17

**Authors:** Fuqiang Cheng, Zahra Foroozan Jahromi, Keqi Wang, Thomas C. Caldwell, Grace Cai, Keilung Choy, Jared Auclair, Jeffrey L. Campbell, Youbo Zhao, Seongkyu Yoon, Sarah W. Harcum, Wei Xie

**Affiliations:** 1 Department of Mechanical and Industrial Engineering, Northeastern University, Boston, MA 02115, USA; 2 Department of Bioengineering, Clemson University, Clemson, SC, USA; 3 Department of Chemical Engineering, University of Massachusetts Lowell, Lowell, MA, USA; 4 Physical Sciences Inc. (PSI), Andover, MA, USA

**Keywords:** induced Pluripotent Stem Cells, Monolayer and Aggregate Cultures, Multi-Scale Mechanistic Modeling, Culture Spatiotemporal Heterogeneity, Cellular Metabolic–Redox Modeling, Multiple Isotope Labeling, Advanced Optical Sensing

## Abstract

Scalable manufacturing of human induced pluripotent stem cells (iPSCs) is essential for industrial-scale production of cell therapies and regenerative medicines. However, the 3D aggregate cultures used in manufacturing exhibit substantial spatial and metabolic heterogeneity compared with the relatively homogeneous monolayer systems used in laboratory studies, complicating mechanistic understanding and predictive metabolic modeling across culture scales. To address this challenge, we developed a modular multiscale mechanistic foundation model that links molecular, cellular, and macroscopic processes while accounting for spatial and metabolic heterogeneity. The framework integrates extracellular culture dynamics, intracellular metabolic fluxes, and cellular redox states by extending a previously established monolayer kinetic network and coupling it with a biological systems-of-systems (Bio-SoS) multiscale model for aggregate cultures, incorporating explicit redox interactions. Systematic monolayer and aggregate experiments—including multiple isotopic tracers, extracellular metabolite profiling, and two-photon optical redox imaging—were used to improve and validate the model. This integrated framework unifies heterogeneous datasets across culture configurations and enables mechanistic interpretation of metabolic and redox responses across heterogeneous culture scales, providing a quantitative foundation for scalable iPSC biomanufacturing.

## 1 Introduction

Human induced pluripotent stem cells (iPSCs) serve as a versatile platform for disease modeling, regenerative medicines, and cell-based therapeutics due to their capacity for indefinite self-renewal and multilineage differentiation [1, 2]. As iPSC-derived products progress toward clinical translation and commercial-scale manufacturing, there is a growing need for robust, scalable culture strategies that preserve metabolic stability, redox homeostasis, and phenotypic consistency [3, 4]. Suspension-based and aggregate-forming culture systems have emerged as particularly promising for large-scale production, as they integrate efficiently with stirred-tank bioreactors and controlled manufacturing environments [5, 4].

However, three-dimensional (3D) aggregate growth introduces substantial spatial heterogeneity in oxygen and nutrient transport, resulting in diffusion limitations, metabolic gradients, and heterogeneous intracellular states across the aggregate radius [6, 7]. Such gradients can significantly alter glycolytic flux, mitochondrial activity, and redox balance, thereby complicating quantitative metabolic characterization and limiting the accuracy of predictive process control in scalable iPSC culture systems [8, 9].

Cellular metabolism plays a central role in regulating iPSC growth and maintaining pluripotency [10]. Compared to differentiated cells, pluripotent stem cells rely heavily on glycolysis, exhibiting elevated glycolytic flux, high lactate production, and reduced dependence on oxidative phosphorylation [11, 9]. Central carbon metabolism through glycolysis and the tricarboxylic acid (TCA) cycle is tightly coupled to intracellular redox reactions involving NAD^+^/NADH, NADP^+^/NADPH, and FAD/FADH_2_, thereby linking extracellular oxygen and nutrient availability to cellular energy production, biosynthetic pathways, and signaling networks.

In 2D static monolayer cultures, cells experience relatively uniform exposure to oxygen and nutrients, enabling their metabolic states to be interpreted under conditions that are effectively homogeneous. In contrast, 3D aggregate cultures impose diffusion limitations that create spatial gradients in oxygen tension and substrate availability. These gradients can drive pronounced shifts in glycolytic activity, mitochondrial flux, and redox balance across the aggregate radius, even when bulk extracellular measurements appear comparable to those of 2D systems.

Because intracellular redox state is an emergent property of metabolic flux distributions and is particularly sensitive to local transport constraints, predictive modeling of scalable iPSC cultures must integrate metabolic regulation with substrate and waste transport and redox dynamics in a unified framework. Only by treating these processes as a tightly interconnected system can model predictions accurately capture the spatial and metabolic heterogeneity inherent to 3D culture environments.

Advances in non-invasive optical sensing have further expanded the ability to integrate metabolic and redox measurements into iPSC culture analysis. Autofluorescence imaging of NAD(P)H and FAD provides label-free assessment of intracellular redox state in living cells [12, 13, 14]. In particular, two-photon excitation (TPE) sensing offers real-time quantification of intracellular metabolites and the NAD(P)H/FAD redox ratio, enabling dynamic monitoring of metabolic state changes within complex culture environments. When implemented in bioreactors, TPE benefits both from its intrinsic optical selectivity and from the high flow dynamics of agitated cultures, producing cytometry-like, single-cell–level metabolic readouts in situ—capabilities that are difficult to achieve in large-scale suspension systems. Consequently, TPE sensing is well suited for aggregate-based iPSC cultures, where destructive intracellular sampling is impractical and spatial heterogeneity complicates the interpretation of conventional biochemical assays.

The proposed multiscale modeling framework for 3D iPSC aggregate cultures links measurable redox dynamics to the underlying intracellular metabolic flux distributions across scales—from 2D cultures commonly used in laboratory studies to 3D aggregate systems recommended for industrial manufacturing. This modular framework supports the construction of mechanistic models for both 2D monolayers and 3D aggregates and can incorporate heterogeneous experimental observations generated across diverse manufacturing systems, including isotopic labeling data, extracellular metabolite profiling, and optical redox sensing. By jointly analyzing isotopic labeling patterns, extracellular metabolite exchange rates, and TPE-derived redox measurements, the framework enables interoperable, scalable, and mechanistically informed iPSC manufacturing.

Quantitative metabolic modeling has been widely applied in mammalian cell cultures to support rational biomanufacturing process development and improve understanding of cellular metabolism [15, 16]. However, translating these approaches to iPSC systems presents additional challenges. Unlike many industrial mammalian production cell lines, which operate under relatively stable productivity-driven objectives, iPSCs exhibit metabolism tightly linked to pluripotency maintenance and redox-sensitive regulatory networks. Furthermore, scalable culture formats such as suspension aggregates introduce diffusion-limited transport of oxygen and nutrients, leading to spatially heterogeneous microenvironments and dynamically varying intracellular metabolic states. These biological and process-specific characteristics complicate direct interpretation of extracellular measurements and highlight the need for mechanistic modeling frameworks tailored to iPSC cultures. Such models must link extracellular culture dynamics with intracellular metabolic flux regulation while accounting for heterogeneity arising from aggregate-scale transport phenomena.

Recent efforts have begun to advance quantitative, iPSC culture modeling frameworks. Wang et al. developed a kinetic metabolic regulatory network model that integrates multiple isotopic tracers to improve intracellular flux analysis in 2D monolayer iPSC cultures, providing a structured representation of glycolysis, TCA cycle activity, and associated regulatory interactions under relatively homogeneous conditions [17]. Recognizing that individual cells operate as complex systems and that cell-to-cell interactions create heterogeneous micro-environments, Zheng et al. introduced a biological systems-of-systems (Bio-SoS) modeling strategy and a modular, multi-scale mechanistic foundational model capable of assembling both 2D monolayer and 3D aggregate cultures [18]. Their framework captures multiscale variability by modeling spatial heterogeneity and population-level dynamics in iPSC aggregates—factors that are essential for controlling quality consistency and for integrating manufacturing processes across scales.

However, existing iPSC culture modeling frameworks either prioritize flux fitting under near-homogeneous monolayer conditions or center on aggregate-scale heterogeneity, yet none are calibrated using systematic experimental observations spanning both monolayer and aggregate cultures. In addition, they do not explicitly account for intracellular redox dynamics. Consequently, quantitative relationships linking nutrient perturbations, intracellular flux redistribution, redox balance, and aggregate growth dynamics remain incompletely characterized.

Building on the kinetic metabolic regulatory network model for monolayer iPSC cultures [17] and the multiscale Bio-SoS framework for 3D aggregate cultures [18], this study further extends and experimentally validates a unified multiscale mechanistic foundation model. First, the intracellular metabolic network is expanded to explicitly represent redox coupling, establishing a direct connection between metabolic flux distributions and the resulting cellular redox dynamics. Second, systematic isotopic experiments performed under static and pyruvate-supplemented culture conditions uncover new metabolic mechanisms that refine our understanding of TCA cycle activity and metabolic–redox interactions. Third, the predictive capability and interoperability of the extended multiscale foundation model are calibrated and evaluated using comprehensive experimental datasets spanning both monolayer and aggregate cultures. Together, these advances provide a quantitative foundation for improved characterization of iPSC metabolic behavior and support the acceleration of scalable, interoperable iPSC manufacturing process development.

## 2 Materials and Methods

### 2.1 Cell Line

K3 induced pluripotent stem cells (iPSCs) were donated by Dr. Stephen A. Duncan at the Medical University of South Carolina. The K3 iPSCs were generated by transient transfection of human foreskin fibroblasts, as previously described [19].

### 2.2 Pre-Culture Conditions

K3 iPSCs were cultured on non–tissue culture–treated 6-well plates (Corning Inc., Corning, NY) coated with 10 *μ*g/mL Vitronectin XF (Catalog #07180; StemCell) according to the manufacturer’s instructions. Cultures were maintained at 37°C in a humidified incubator with 5% CO_2_ using Essential 8 Flex (E8 Flex) medium (Thermo Fisher Scientific, Waltham, MA). The working volume was 2 mL per well. Cells were passaged every 3 days at split ratios of 1:6 or 1:12 depending on confluency.

For passaging, cells were dissociated using Gibco StemPro Accutase Cell Dissociation Reagent (Thermo Fisher Scientific) for 2 min at 37°C, followed by washing with phosphate-buffered saline (PBS) without calcium or magnesium (Corning Inc.). Detached cells were centrifuged at 500 × *g* for 5 min and resuspended in fresh E8 Flex supplemented with 10 *μ*M Y-27632, a rho-associated coiled-coil containing protein kinase inhibitor (ROCKi; STEMCELL Technologies, Vancouver, Canada). After 24 h, the medium was replaced with E8 Flex without ROCKi.

### 2.3 Static Pyruvate Cultures: Growth and Parallel Labeling

For growth and isotope labeling studies, K3 iPSCs were seeded at a density of 1 × 10^4^ cells/cm^2^ in E8 Flex supplemented with 10 *μ*M ROCK inhibitor to promote attachment and reduce apoptosis. After 24 h, a complete media exchange was performed. One of four defined growth media (2 glucose levels × 2 lactate levels) was added to parallel 6-well plates and T75 flasks. An overview of the experimental design, including initial concentrations of glucose, glutamine, lactate, and pyruvate, is provided in Table 1. E8 Flex was prepared without glucose to enable controlled adjustment of glucose levels. For isotope-labeling experiments, 4 mM [U-^13^C_3_] pyruvate (99% enrichment; Cambridge Isotope Laboratories, Tewksbury, MA) was used.

### 2.4 Aggregate Cultures

Suspension aggregate cultures were established following previously reported protocols [20]. Briefly, static culture cells were dissociated using Accutase, and the reaction was neutralized with pre-warmed medium. Cells were centrifuged at 200 × *g* for 4 min at 25°C, and the supernatant was discarded. The cell pellet was gently resuspended in approximately 10 mL of pre-warmed medium to generate a single-cell suspension. Cells were counted and resuspended in E8 Flex supplemented with 10 *μ*M ROCKi prior to aggregate formation.

### 2.5 Analytical Methods

#### 2.5.1 Cell Concentration and Metabolite Analysis

Cell concentration and extracellular glucose, lactate, pyruvate, ammonia, and amino acid concentrations were measured for static pyruvate and aggregate cultures using methods summarized in Table 1 and in accordance with standard manufacturing protocols. For aggregate cultures, a 1 mL sample was collected and aggregates were pelleted by centrifugation at 500 × *g* for 5 min. The supernatant was retained for metabolite and amino acid analysis. The cell pellet was subsequently dissociated using Gibco StemPro Accutase and resuspended in 1 mL of fresh medium for cell counting and viability assessment.

#### 2.5.2 Aggregate Size Distribution

Aggregate size distributions were measured daily using one shaker flask per condition. To assess aggregate morphology and size, 1–2 mL of culture was transferred into 12-well plates or petri dishes and allowed to settle prior to imaging. Brightfield images were acquired using an Olympus microscope equipped with a digital camera (cellSens Standard 3.2). Aggregate diameters and counts were quantified using ImageJ (NIH). Images were background-subtracted, converted to binary format, and filtered by circularity to exclude debris and non-aggregate objects. Size distributions were calculated from more than 50 aggregates per sample using the *Analyze Particles* function.

#### 2.5.3 Enzymatic Redox Assay

Intracellular concentrations of NADH and NAD^+^ were quantified using the EnzyChrom NAD^+^/NADH Assay Kit (BioAssay Systems, Hayward, CA) following the manufacturer’s protocol. One shaker flask was harvested per time point, and 1 mL of aggregates was enzymatically dissociated using Accutase as described above. Fluorescence measurements were obtained using a BioTek Synergy H1 microplate reader, with data acquisition and analysis performed using Gen5 software (BioTek Instruments, Winooski, VT). The redox ratio was calculated as $\frac{{NAD}^{+}}{{NAD}^{+}+NADH}$ using normalized fluorescence intensities.

#### 2.5.4 TPE Redox Measurement

For optical redox measurements using the two-photon excitation (TPE) system (Prototype-002, PSI), the contents of the shaker flask were transferred to a beaker and placed on a pre-warmed stirring platform. Autofluorescence signals from NAD(P)H and FAD were recorded in real time at the single cell level. The optical redox ratio was calculated as $\frac{\text{FAD}}{\text{FAD}+\text{NAD}(P)H}$, and redox estimates were obtained using PSI’s proprietary data analysis software. This redox ratio is widely used as an optical indicator of cellular metabolic state derived from endogenous fluorescence signals [21]. It reflects the balance between oxidized flavin cofactors and reduced nicotinamide cofactors and has been shown to correlate with cellular metabolic shifts.

Although the enzymatic assay quantifies the ratio of NAD^+^ to total nicotinamide cofactors, while the optical measurement captures the fluorescence ratio of FAD and NAD(P)H, both metrics represent the balance between oxidized and reduced cofactors associated with cellular metabolic activity [21]. Agreement between trends from the enzymatic assay and the TPE-derived optical redox ratio therefore supports the use of the TPE sensor as a non-destructive method for monitoring intracellular redox dynamics.

## 3 Experiment Results and Discussion

### 3.1 Growth Characteristics of Static Pyruvate and Aggregate Cultures

To advance the characterization of iPSC metabolism and redox regulation, this study employed isotopically labeled pyruvate across combinations of high and low glucose (HG, LG) and high and low lactate (HL, LL) conditions. This investigation builds upon our previously established iPSC monolayer culture system and comprehensive isotope-labeling experiments. Odenwelder et al. [22] evaluated iPSC metabolism under four media formulations using labeled glucose and glutamine to resolve intracellular fluxes. Although labeled lactate was also assessed, the resulting intracellular enrichment was insufficient for inclusion in metabolic flux analysis. Motivated by this limitation, we hypothesized that labeled pyruvate would provide more robust intracellular labeling patterns.

Preliminary experiments were conducted to determine pyruvate tolerance, revealing that concentrations of 20.0 mM and 10.0 mM completely inhibited cell growth. Consequently, 4.0 mM pyruvate was selected for the isotope labeling experiments in high glucose low lactate (HGLL), high glucose high lactate (HGHL), low glucose high lactate (LGHL), and low glucose low lactate (LGLL) conditions.

**Figure 1:**
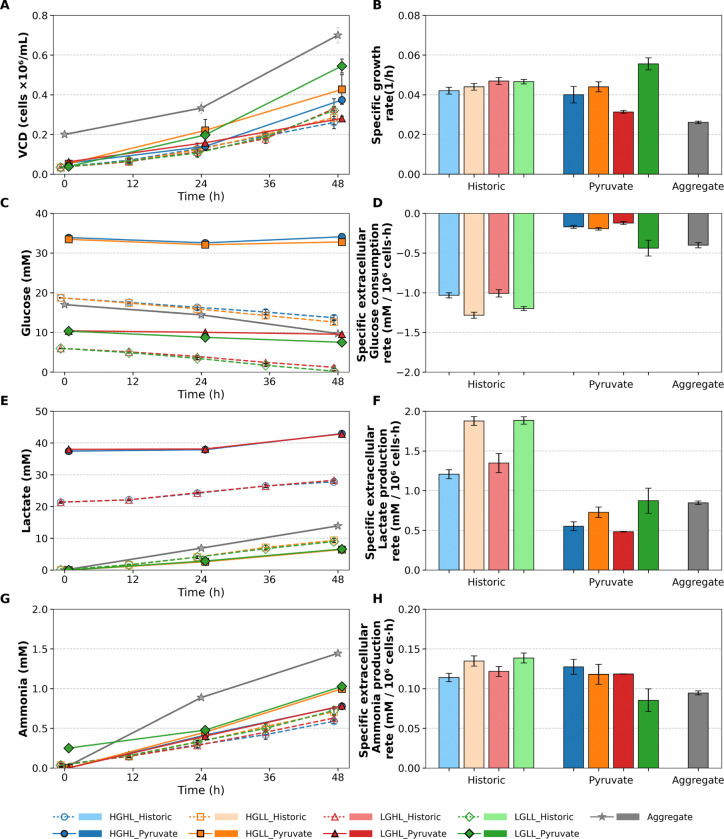
Cell growth, glucose, lactate, and ammonia profiles and rates for Historic Static, Static Pyruvate, and Aggregate culture conditions. (A) Viable cell density (VCD) profiles. (B) Specific growth rates. (C) Glucose concentration profiles. (D) Specific glucose consumption rates. (E) Lactate concentration profiles. (F) Specific lactate production rates. (G) Ammonia concentration profiles. (H) Specific ammonia production rates. Error bars represent the standard deviation across biological replicates. For clarity, data points corresponding to Historic Static conditions are slightly shifted to the left of each sampling time, whereas Static Pyruvate conditions are slightly shifted to the right to facilitate visual distinction of overlapping measurements.

**Figure 2:**
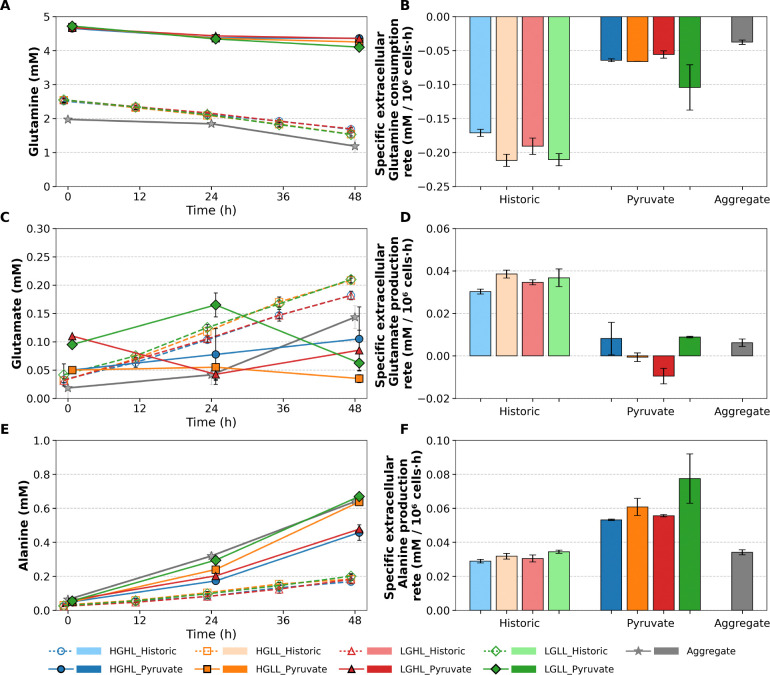
Glutamine, glutamate, and alanine profiles and rates for Historic Static, Static Pyruvate, and Aggregate culture conditions. (A) Glutamine concentration profiles. (B) Specific glutamine consumption rates. (C) Glutamate concentration profiles. (D) Specific glutamate production rates. (E) Alanine concentration profiles. (F) Specific alanine production rates. Error bars represent the standard deviation across biological replicates. For clarity, data points corresponding to Historic Static conditions are slightly shifted to the left of each sampling time, whereas Static Pyruvate conditions are slightly shifted to the right to facilitate visual distinction of overlapping measurements.

The growth characterization of the Static Pyruvate cultures was compared to the Historic Static cultures [22] as well as Aggregate cultures in standard E8 Flex media. Figs. 1 and 2 compare the growth and metabolic profiles of these three experimental setups. The initial glucose, glutamine, and pyruvate concentration for Static Pyruvate cultures were significantly higher than that used for the Historic Static cultures and Aggregate cultures, and the high lactate conditions were also significantly higher for the Static Pyruvate cultures. Interestingly, the growth rates for all culture conditions were similar; however, the glucose consumption rate was significantly lower for the Static Pyruvate cultures due to the presence of pyruvate.

**Figure 3:**
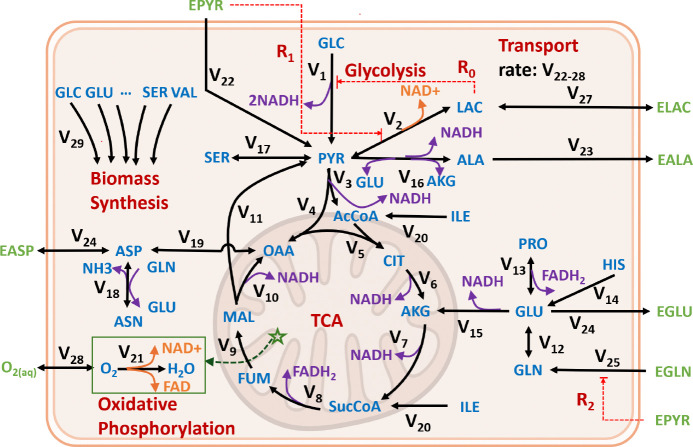
Metabolic network for iPSC including regulatory reactions. Intracellular metabolites are shown in blue, while extracellular metabolites are shown in green and denoted with an “E” prefix. Reaction fluxes are represented by black arrows, with transport reactions connecting intracellular and extracellular species. Energy-generating steps are highlighted in purple, whereas energy-consuming steps are indicated in orange. Oxidative phosphorylation in the mitochondria, associated with NADH oxidation and oxygen consumption, is indicated by the green star. Reactions involving NAD^+^/NADH and FAD/FADH_2_ are explicitly annotated to capture cellular redox balance. The network integrates glycolysis, the TCA cycle, oxidative phosphorylation, amino acid metabolism, and biomass synthesis. EPYR in Static Pyruvate culture represents both labeled and unlabeled extracellular pyruvate.

Additionally, glutamine consumption and glutamate production ratio were significantly lower for the Static Pyruvate cultures, which may also be due to pyruvate inhibition [23]. Alanine production was higher for the Static Pyruvate Cultures compared to the Historic Static cultures and Aggregate cultures, likely due to direct alanine synthesis from pyruvate; see Fig. 3. Several amino acids exhibited reduced consumption rates in Static Pyruvate Cultures compared with Historic Static Cultures. In general, amino acid inhibition mechanisms due to high pyruvate concentration have not been systematically studied in the literature and require further investigation.

Furthermore, lower glucose consumption and lactate production were observed in aggregate cultures, likely due to lower oxygen availability throughout the aggregates [6, 7]. In the present study, oxygen transport was not explicitly modeled because dissolved oxygen levels were maintained within a non-limiting range in the bulk medium, and the available experimental data were insufficient to resolve intra-aggregate oxygen gradients required for reliable parameterization. Glutamine consumption was also lower in aggregate cultures compared with historic static cultures, while glutamate production was reduced and more similar to that observed in static pyruvate cultures. One possible reason is the inhibitory effect of pyruvate on glutamine utilization [23]. Finally, ammonia production was similar across all three culture conditions, potentially due to counterbalancing effects of reduced glutamine consumption and glutamate production; see $V_{12}$ in Fig. 3.

### 3.2 Isotopic Analysis of Static Pyruvate Cultures

To improve isotopic resolution of metabolic fluxes, 4.0 mM [U-^13^C_3_] pyruvate was added to the Static Pyruvate cultures for the labeling experiments. This tracer was incorporated into the E8 Flex medium, yielding a final total pyruvate concentration of 4.4 mM. For consistency, the unlabeled control cultures used for growth and metabolite measurements also received 4.0 mM pyruvate. This additional pyruvate supplement was applied across all four glucose/lactate conditions (HGLL, HGHL, LGHL, and LGLL).

**Figure 4:**
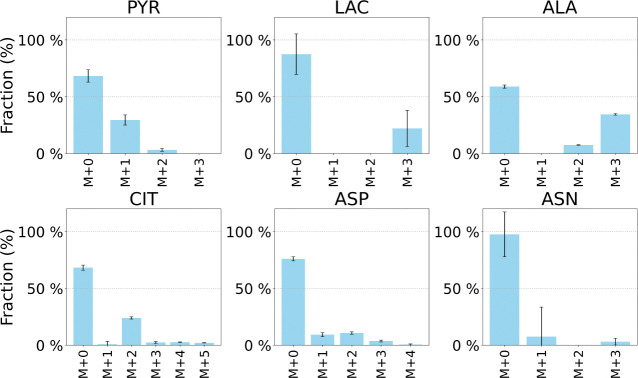
Intracellular MID data for the Static Pyruvate HGLL culture condition at 48 h. MID data were corrected for natural isotopic abundance. Metabolite abbreviations are: PYR (pyruvate), LAC (lactate), ALA (alanine), CIT (citrate), ASP (aspartate), and ASN (asparagine). A complete list of abbreviations is provided in Supplementary Materials Table S1. The intracellular MIDs for the LGLL, HGLL, and LGHL static pyruvate cultures are provided in the Supplementary Materials (Fig. S1–S3).

Across all four Static Pyruvate culture conditions, only low levels of ^13^C enrichment were detected in glucose and in the TCA-cycle intermediates *α*-ketoglutarate, fumarate, succinate, and malate, likely due to detection limitations. For this reason, these metabolites were omitted from the Mass isotopomer distributions ((MIDs) shown in Fig. 4 for clarity. In contrast, substantial ^13^C labeling was observed in lactate and alanine. Previous studies indicate that lactate is derived exclusively from cytosolic pyruvate, whereas alanine can originate from both cytosolic and mitochondrial pools [11, 24, 25]. These observations suggest that most of the exogenously supplied pyruvate remained in the cytosol, with limited transport into the mitochondria—consistent with the minimal labeling observed in TCA intermediates. Additionally, unlabeled glutamine and glutamate supplied in the medium continue to feed the TCA cycle, further diluting ^13^C enrichment. The unlabeled fractions of lactate and alanine likely arise from unlabeled glucose and, to a lesser extent, from the residual unlabeled pyruvate present in the medium.

### 3.3 Aggregate Size and Dynamic Distribution

**Figure 5:**
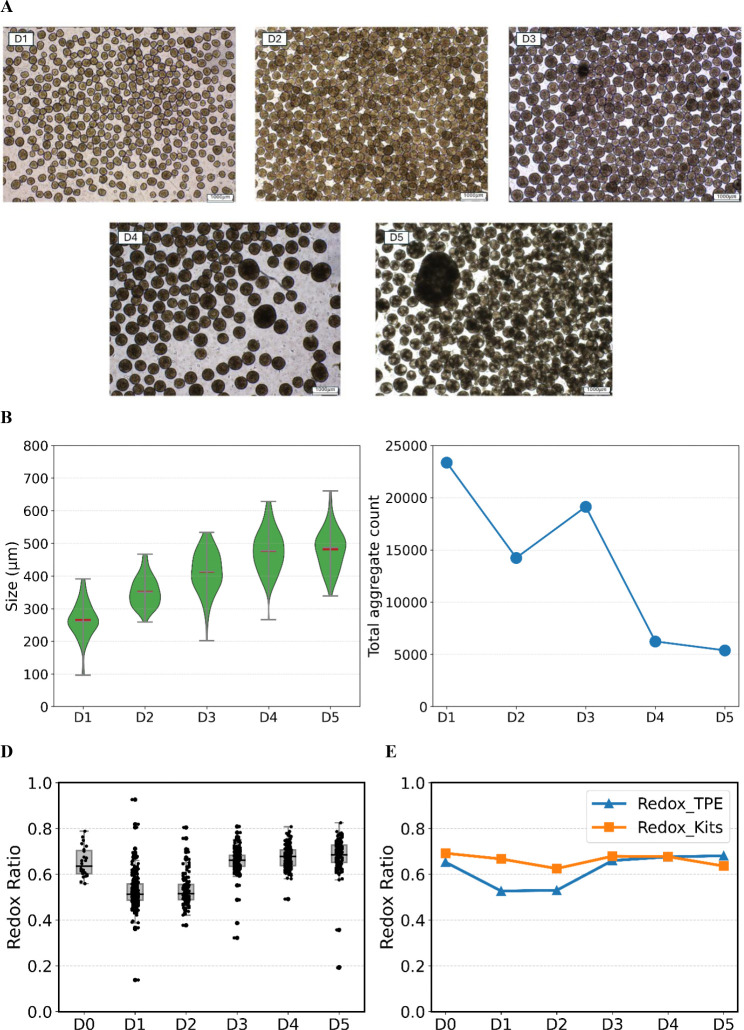
Aggregate growth, size distribution, and redox dynamics of iPSC aggregate cultures. (**A**) Representative brightfield images of iPSC aggregates from Day 1 to Day 5 illustrating aggregate growth and morphological evolution over time. The scale bar (bottom right) corresponds to 1000 *μ*m. (**B**) Violin plots showing the distribution of iPSC aggregate sizes across five days of culture. Green violins represent the kernel density of aggregate size measurements at each time point. Red horizontal bars denote the mean aggregate size, while gray horizontal bars indicate the median. Gray vertical bars and whiskers show the minimum and maximum observed aggregate sizes. Day 3 corresponds to measurements taken prior to feeding, and aggregate size distributions were similar before and after feeding. (**C**) Total aggregate counts measured from Day 1 to Day 5. (**D**) Distribution of redox ratios measured in aggregate cultures from Day 0 to Day 5 using the TPE sensor. Boxplots show the median and interquartile range, with whiskers indicating the full data range; individual replicate measurements are overlaid as points. (**E**) Temporal trends in redox ratios measured by a non-destructive TPE-based method, FAD/(FAD+NAD(P)H), and by an offline enzymatic assay, NAD^+^/ (NAD^+^+NADH), demonstrating consistent redox dynamics across measurement modalities. The TPE-based values represent averages calculated over both the measurement period and the analyzed cell population.

To support multiscale mechanistic modeling of iPSC aggregate cultures, changes in aggregate size distributions were quantified over the 5-day culture period. Fig. 5A shows representative images of aggregates over the 5-day culture period, while Fig. 5B presents the corresponding violin plots of size distributions and Fig. 5C summarizes aggregate counts. As expected, aggregate size increased substantially over time, from 265.4±5.4 *μ*m to 481.4±6.2 *μ*m on day 5. The images in Fig. 5A illustrate this progression: early aggregates (D1–D2) are smaller and relatively uniform, reflecting initial cluster formation, whereas aggregates at later stages (D3–D5) appear larger and darker, consistent with increased cell density and ongoing proliferation. The violin plots in Fig. 5B corroborate this trend, showing a clear upward shift in size distributions and increasing spread over time, indicating greater heterogeneity in aggregate growth.

In contrast, the total number of aggregates decreases over the culture period (Fig. 5C), particularly after day 3. This inverse relationship between aggregate size and aggregate number suggests that growth is driven not only by intracellular proliferation but also by aggregate–aggregate interactions, such as merging or coalescence. As smaller aggregates fuse or expand into larger structures, the culture transitions from many small aggregates to fewer, larger ones.

This structural evolution has significant functional consequences. With increasing aggregate size, diffusion limitations in oxygen and nutrient transport become more severe, giving rise to substantial spatial and metabolic heterogeneity. Accurately capturing this heterogeneity is essential for multiscale mechanistic foundation modeling to systematically represent iPSC cultures across scales.

### 3.4 Redox Measurements for Aggregate Culture

To quantify redox levels in the aggregate cultures, two complementary methods were used. The enzymatic assay requires cell disruption, after which NAD^+^ and NADH concentrations are measured and used to calculate the redox ratio: $\frac{{NAD}^{+}}{{NAD}^{+}+NADH}$. In contrast, the two-photon excitation (TPE) sensor enables nondestructive measurement of FAD and NAD(P)H autofluorescence signals, from which the optical redox ratio is calculated as: $\frac{\text{FAD}}{FAD+NAD(P)H}$. Figs. 5D and E show the redox ratios for the aggregate cultures over the 5-day period. Overall, the redox dynamics captured by the real-time TPE sensor aligned well with the offline enzymatic measurements, supporting the sensor’s reliability for monitoring metabolic state in iPSC aggregates.

## 4 Multi-Scale Model Development

Based on experimentally characterized iPSC growth kinetics, extracellular metabolite profiles, MID data, intracellular redox measurements, and aggregate size distributions collected from both static and aggregate cultures, we developed a multiscale mechanistic modeling framework. This framework builds on our previously established single-cell metabolic model and extends it to explicitly incorporate intracellular redox dynamics and regulatory interactions associated with pyruvate metabolism. Using this enhanced single-cell model as the core module, we further integrated population-balance and reaction–diffusion formulations to represent aggregate formation, transport processes, and intra-aggregate nutrient gradients. The resulting modular framework was calibrated and validated against the full suite of experimental datasets, enabling quantitative prediction of iPSC metabolic behavior across distinct culture configurations. Details of the enhanced single-cell metabolic model are presented in Section 4.1, followed by the development of the multiscale aggregate model in Section 4.2.

### 4.1 Single-Cell Metabolic Model Extension

Building on the results reported in [17], this section extends the mechanistic single-cell metabolic model developed for 2D monolayer iPSC cultures. This enhanced model serves as the core computational module within the modular multiscale framework for 3D aggregate cultures introduced in Section 4.2. Because 2D monolayer cultures experience relatively homogeneous environmental conditions, the associated single-cell metabolic model—schematically illustrated in Fig. 3—builds on previously established frameworks [16, 17]. Relative to the prior iPSC metabolic regulatory network model [17], the present formulation explicitly incorporates intracellular redox reactions through the inclusion of redox cofactors (NAD^+^/NADH and FAD/FADH_2_) and a simplified representation of oxidative phosphorylation. These extensions enable the model to capture the experimentally observed coupling between central carbon metabolism and cellular redox balance.

To represent the inhibitory effect of lactate on glucose uptake, a lactate-dependent regulatory element was incorporated following Odenwelder et al. [22] and is denoted as R0 in Fig. 3. In addition, motivated by experimental observations from the present study, two additional regulatory mechanisms (R1 and R2) were introduced to account for the effects of pyruvate on cellular metabolism.

#### R1: Pyruvate inhibition on lactate production [26] (R1 in Fig. 3).

Recent experimental evidence demonstrates that elevated extracellular pyruvate can inhibit lactate dehydrogenase (LDH) activity in living cells through an MCT1-dependent mechanism [26]. Pyruvate and lactate share the same monocarboxylate transporters (MCTs), and high extracellular pyruvate concentrations increase intracellular pyruvate levels through MCT1-mediated transport. The resulting rise in intracellular pyruvate shifts the LDH equilibrium, suppressing the forward conversion of pyruvate to lactate by perturbing the NADH/NAD^+^ redox balance and competing with lactate export. Consequently, excess pyruvate reduces net lactate production and redirects carbon flux toward mitochondrial oxidation pathways.

To capture this regulatory effect, an inhibitory term dependent on extracellular pyruvate concentration (EPYR) is incorporated into the LDH rate expression:

$$\begin{array}{ll} v(LDH)= & v_{maxfLDH}\frac{PYR}{K_{mPYR}\;+\;PYR}\times \frac{NADH/NAD}{K_{mNADHtoNAD}\;+\;NADH/NAD}\times \frac{K_{iEPYR}}{K_{iEPYR}\;+\;EPYR}\\ & \;-v_{maxrLDH}\frac{LAC}{K_{mLAC}\;+\;LAC}\times \frac{NAD/NADH}{K_{mNADtoNADH}\;+\;NAD/NADH}. \end{array}$$

This term reduces the effective LDH activity as extracellular pyruvate accumulates, capturing the experimentally observed suppression of lactate production under high-pyruvate conditions.

#### R2: Pyruvate inhibition on glutamine consumption [23] (R2 in Fig. 3).

Glutamine is a major anaplerotic substrate that replenishes tricarboxylic acid (TCA) cycle intermediates and supports biosynthesis in proliferating cells. Previous studies have shown that glutamine oxidation becomes particularly important when mitochondrial pyruvate transport is limited, enabling cells to sustain TCA cycle activity in the absence of sufficient pyruvate-derived carbon [23]. Under these conditions, glutamine-derived carbon is converted to *α*-ketoglutarate and fuels downstream TCA cycle reactions. Conversely, when pyruvate availability is high, mitochondrial pyruvate oxidation can directly provide acetyl-CoA to the TCA cycle, reducing the reliance on glutamine as an anaplerotic substrate. To capture this metabolic regulation, an inhibitory term dependent on extracellular pyruvate concentration (EPYR) is incorporated into the glutamine consumption rate expression:

$$v(GLNS)=v_{maxfGLNS}\frac{GLN}{K_{mGLN}\;+\;GLN}\times \frac{K_{iEPYR}}{K_{iEPYR}\;+\;EPYR}-v_{maxrGLNS}\frac{GLU}{K_{mGLU}\;+\;GLU}\times \frac{{NH}_{4}}{K_{mNH4}\;+\;{NH}_{4}}.$$

Overall, the single-cell metabolism model contains 29 reactions, each associated with a corresponding flux rate. All reactions are listed in the Supplementary Materials (Table S2), along with the corresponding flux-rate expressions in Table S3. In addition to incorporating the expanded redox network, the model extends the previous iPSC mechanistic framework [17] by introducing a biomass-associated growth rate (see Equation (29) in Table S3). The stoichiometric coefficients for amino acid consumption in the biomass equation were derived from the amino acid composition reported in [27], enabling a more accurate representation of iPSC growth kinetics and amino acid consumption.

### 4.2 Multiscale Model Development

Building on the single-cell metabolic model described in Section 4.1, a multiscale mechanistic framework—referred to as the System-of-Systems (SoS) model [18]—was employed to describe iPSC aggregate culture behavior while accounting for cell-to-cell interactions and spatiotemporal heterogeneity. This multiscale framework consists of the following interconnected modules:

1. **Single-Cell Metabolism Model:** Characterizes the coupled metabolic and redox networks governing cellular responses to spatially heterogeneous microenvironments.
2. **Population Balance Model:** Represents cell-to-cell interactions to capture the dynamic evolution of iPSC aggregate size distributions in bioreactor cultures.
3. **Reaction-Diffusion Model:** Characterizes the intra-aggregate diffusion dynamics and describe spatial nutrient and metabolite gradients within aggregates.

The developed multiscale mechanistic model, with its modular design, enables the integration of heterogeneous data from both monolayer and aggregate cultures while capturing metabolic and spatial heterogeneity. This framework can guide robust culture optimization, improve production consistency and interoperability, and accelerate iPSC manufacturing scale-up.

#### 4.2.1 Cell–Cell Interactions & Aggregation Dynamics

To characterize the formation and evolution of iPSC aggregates, we employed a population balance model (PBM) [28, 29, 6] to describe cell–cell interactions and aggregation dynamics during aggregate culture. In these systems, individual cells and small clusters progressively collide, adhere, and proliferate, giving rise to multicellular aggregates whose size distribution changes over time (see Section 3.3). These dynamics arise from the interplay of biological processes—such as cell adhesion, proliferation, and extracellular matrix deposition—and physical mechanisms including aggregate–aggregate coalescence. The PBM offers a quantitative framework for capturing these effects by tracking the time-dependent evolution of the aggregate size distribution.

Let ϕ(x,t) denote the number density of aggregates of size x at time t. The temporal evolution of the size distribution is governed by the population balance equation:

(1) $$\frac{\partial \phi (x,\;t)}{\partial t}=\frac{1}{2}\int_{x_{0}}^{x}\phi \left(x_{c},t\right)\phi \left(x^{\prime },t\right)K\left(x_{c}|x^{\prime }\right)dx^{\prime }-\int_{x_{0}}^{\infty }\phi (x,t)\phi \left(x^{\prime },t\right)K\left(x|x^{\prime }\right)dx^{\prime }-\frac{\partial }{\partial x}\left[\phi (x,t)\frac{\partial x}{\partial t}\right].$$

The three terms on the right-hand side represent the fundamental mechanisms shaping aggregate size evolution. The first term captures the *formation* of aggregates of size x through the merger of two smaller clusters of sizes $x^{\prime }$ and $x_{c}=x-x^{\prime }$. The product $\phi \left(x_{c},t\right)\phi \left(x^{\prime },t\right)$ reflects their interaction frequency. The second term describes the *loss* of aggregates of size x as they combine with other clusters to form larger aggregates. The third term accounts for size *growth* driven by cell proliferation within aggregates, modeled as $\frac{\partial x}{\partial t}=\alpha_{G}xlog\left(\frac{M}{x}\right)$, where $\alpha_{G}$ is proportional to the cellular growth rate and M represents the maximum attainable aggregate size.

The aggregation kernel governing interactions between aggregates is defined as

$$K\left(x|x^{\prime }\right)=k\cdot exp\left(-k_{1}{\left(\frac{x\;+\;x^{\prime }}{2}\right)}^{a}\right){\left(x^{\frac{1}{3}}+x^{\prime \frac{1}{3}}\right)}^{7/3}$$

following [28]. In this formulation, the parameter k represents the baseline hydrodynamic collision frequency and serves as the overall aggregation rate constant. The exponential term captures the size-dependent decline in adhesion probability due to surface-related constraints; here $k_{1}$ controls the magnitude of this size-dependent inhibition, and a determines the sensitivity of adhesion efficiency to aggregate size. The geometric factor ${\left(x^{\frac{1}{3}}+x^{\prime \frac{1}{3}}\right)}^{7/3}$ encodes how the effective collision cross-section scales with the sizes of the interacting aggregates, reflecting classical geometric considerations in cluster–cluster aggregation.

#### 4.2.2 Coupled Reaction and Diffusion Dynamics

To characterize spatial gradients of nutrients and metabolites within aggregates, a reaction–diffusion model [30, 6] was employed to describe the transport and consumption of extracellular metabolites. Aggregates are approximated as spherical cell clusters with radial symmetry. The concentration of metabolite i within the aggregate satisfies

$$\frac{\partial c_{i}}{\partial t}=\frac{D_{i}}{r^{2}}\frac{\partial }{\partial r}\left(r^{2}\frac{\partial c_{i}}{\partial r}\right)+\rho_{i}(c,s),$$

where $c_{i}(r,t)$ denotes the concentration of metabolite i at radial position r∈[0,R] and time t, with R denoting the aggregate radius. Here, $D_{i}$ is the effective diffusion coefficient of metabolite i within the aggregate, and $\rho_{i}(c,s)$ represents the local metabolic reaction rate determined by the intracellular metabolic model (see Section 4.1), where c denotes the vector of extracellular metabolite concentrations and s represents the intracellular metabolic states governing cellular uptake and secretion.

Through coupling with the single-cell metabolic model, extracellular nutrient and metabolite gradients modulate intracellular metabolic fluxes, while cellular metabolism simultaneously reshapes the aggregate microenvironment. Together, the population balance and reaction–diffusion modules link aggregate-scale transport and population dynamics with intracellular metabolic behavior. This modular multiscale framework enables integration of heterogeneous observations from both monolayer and aggregate cultures and provides a mechanistic basis for analyzing metabolic heterogeneity, diffusion limitations, and aggregate growth dynamics in iPSC bioprocesses.

## 5 Model Fitting and Validation

The multiscale foundation model integrates three components: (i) a single-cell mechanistic model, (ii) a population balance model describing the aggregation process, and (iii) a diffusion–reaction module that captures intra-aggregate transport and reaction dynamics of nutrients and metabolites. We iteratively leveraged heterogeneous measurements from both 2D monolayer and 3D aggregate cultures to calibrate the individual modules of the multiscale mechanistic foundation model, as detailed below.

### (1) Single-cell metabolism model fitting.

All eight monolayer datasets (four Historic Static conditions [22] and four Static Pyruvate conditions) were used to fit the cell metabolic kinetic model parameters described in Table S4. Parameter estimation was performed by minimizing the mean squared error (MSE) between model predictions and experimental measurements:

(2) $$MSE=\frac{1}{KI}\sum_{k=1}^{K}\sum_{i=1}^{I}\frac{1}{T}\sum_{t\in 𝒯}{\left(y_{i,t}^{\left(k\right)}-{\overset{ˆ}{y}}_{i,t}^{\left(k\right)}\right)}^{2},$$

where $y_{i,t}^{\left(k\right)}$ is the experimental measurement of state variable i at time t for dataset k, and ${\overset{ˆ}{y}}_{i,t}^{\left(k\right)}$ is the corresponding model prediction. The measurement time sets 𝒯={12,24,36,48} or 𝒯={24,48} depend on the specific experimental study k. Here, K denotes the total number of datasets, I is the total number of state variables measured, and T the total number of measurements. The estimated single-cell mechanistic parameter values are provided in the Supplementary Materials (Table S4).

### (2) Population balance model and training.

Aggregate size measurements were used to calibrate a population balance model (PBM) that characterizes aggregation dynamics. The predicted aggregate distribution $\overset{ˆ}{\phi }(x,t)$ and the empirical observations on iPSC aggregate size distribution ϕ(x,t) from day 1 to day 3 were compared by minimizing the KL divergence:

$$min\;D_{KL}(\phi \|\overset{ˆ}{\phi })=min\sum_{t\in 𝒯}\phi \left(x,t\right)\log\left(\frac{\phi \left(x,\;t\right)}{\overset{ˆ}{\phi }\left(x,\;t\right)}\right)dx,$$

where 𝒯={24,48,72} representing days 1 to 3. Only the first three days were used for model fitting because cell viability declined noticeably after day 3. Non-viable cells distort the aggregation dynamics and are not accounted for in the current population-balance model, potentially biasing parameter estimates.

### (3) Reaction-diffusion module and training.

The reaction–diffusion module describes the transport and consumption of nutrients and metabolic wastes, enabling quantification of the spatially heterogeneous microenvironment arising from cell–cell interactions within aggregates. This module models the spatiotemporal evolution of key extracellular components, including nutrients and metabolites such as glucose and lactate. Aggregates are approximated as spherical cell clusters with radial symmetry, assuming isotropic diffusion along the radial direction. Detailed model implementation are provided in [18].

For training the reaction–diffusion module, the diffusion coefficients of each substrate (initial values provided in Supplementary Table S5) were perturbed to minimize the mean squared error (MSE) between model predictions and extracellular metabolite measurements from the aggregate culture dataset. The MSE loss function follows Equation (2), with K=1 corresponding to a single dataset and the measurement time set T=24,48,72.

## 6 Model Prediction Performance

Following model development and calibration, the predictive capability of the proposed framework was evaluated across multiple biological scales and culture configurations. Model performance was assessed by comparing predictions against experimental measurements of extracellular metabolite dynamics, intracellular MIDs, redox responses, aggregate size evolution, and aggregate-scale metabolic behavior. These analyses evaluated both the quantitative agreement between model predictions and experimental observations and the framework’s ability to generalize across manufacturing scales exhibiting different degrees of heterogeneity.

Subsections 6.1 and 6.2 present prediction results for 2D monolayer and 3D aggregate cultures, respectively. For monolayer systems, the evaluation focuses on extracellular metabolite dynamics, isotopic labeling behavior, and intracellular redox responses. For aggregate cultures, the assessment further examines the ability of the multiscale framework to reproduce aggregate growth, size distribution evolution, and extracellular metabolic profiles arising from diffusion-limited microenvironments and cell–cell interactions.

### 6.1 Monolayer Model Prediction Performance

This section evaluates the predictive performance of the single-cell metabolic model under monolayer culture conditions. Because monolayer systems provide a relatively homogeneous extracellular environment, they offer a controlled setting for assessing the model’s ability to capture intracellular metabolic regulation. Model performance is examined from three complementary perspectives: (i) prediction of extracellular metabolite dynamics under diverse nutrient conditions (Section 6.1.1), (ii) reproduction of intracellular mass isotopomer distributions from tracer experiments (Section 6.1.2), and (iii) prediction of intracellular redox responses to environmental perturbations (Section 6.1.3). Together, these analyses provide a systematic validation of the single-cell metabolic model presented in Section 4.1 before extending the evaluation to the spatially heterogeneous aggregate cultures described in Section 4.2.

#### 6.1.1 Extracellular Metabolite Prediction

To evaluate the model’s robustness and predictive performance across different extracellular environments, a leave-one-out cross-validation strategy was employed. In each iteration, the model was trained on seven of the eight culture conditions—four Historic Static conditions [22] and four Static Pyruvate conditions—and tested on the remaining condition. This procedure enabled us to test the model’s ability to generalize to previously unseen environmental settings, including new combinations of glucose, lactate, and pyruvate concentrations.

The cross-validation results across eight culture conditions demonstrated that the model has robust prediction performance under diverse nutrient and metabolite environments. For the Historic Static cultures, the model consistently captured key metabolic behaviors such as glucose and glutamine consumption, lactate and ammonium accumulation, and amino acid dynamics, as shown in the Supplementary Materials (Fig. S4–S7). Particularly strong agreement was observed in HGHL, LGLL, and LGHL conditions, while the HGLL condition showed minor deviations—primarily in the alanine profile (EALA). Nonetheless, overall temporal trends were well reproduced, indicating that the model effectively generalizes to unseen conditions and accurately captures metabolic shifts driven by nutrient depletion or byproduct accumulation.

**Figure 6:**
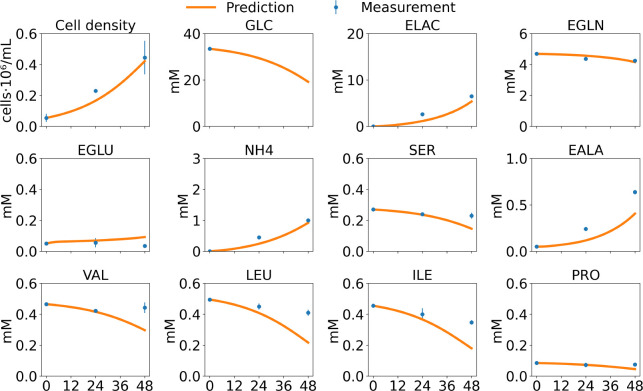
Dynamic model cross-validation for the HGLL static pyruvate condition. The model was trained using all Historic Static culture datasets and the remaining Static Pyruvate culture conditions and then used to predict the HGLL case. Error bars represent the standard deviation observed across replicate HGLL static pyruvate cultures. Cross-validated predictions for all static pyruvate conditions are provided in the Supplementary Materials (Fig S2–S4).

For the Static Pyruvate cultures, the model showed slightly reduced predictive performance compared to the Historic Static conditions, particularly in capturing alanine dynamics. This discrepancy may arise from unmodeled compartmentalization between cytosolic and mitochondrial pyruvate pools, which influence alanine biosynthesis through distinct pathways. Model predictions for the HGLL Static Pyruvate condition are shown in Fig. 6, with results for the remaining three conditions provided in the Supplementary Materials (Figs. S8–S10). In addition, the model substantially underestimated cell density in the LGLL condition, which consequently reduced the accuracy of metabolite predictions—even when qualitative trends appeared visually consistent. These limitations may also be partially attributed to the smaller number of biological replicates in the Static Pyruvate datasets (2 replicates) relative to the Historic Static cultures (6 replicates), increasing estimation uncertainty. Despite these challenges, the model still demonstrated strong predictive capability across pyruvate supplementation scenarios, supporting its applicability to both standard and modified iPSC culture conditions.

#### 6.1.2 MID Validation and Prediction

**Figure 7:**
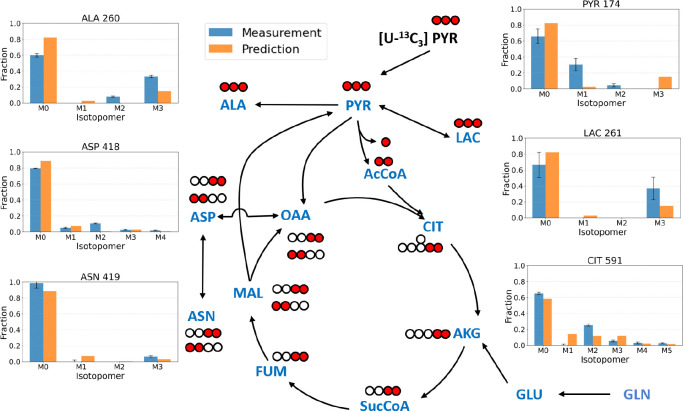
Model prediction of mass isotopomer distributions for glycolytic and TCA cycle metabolites in the Static Pyruvate culture (HGLL) with [U-^13^C_3_]pyruvate as the tracer. The central schematic illustrates representative carbon transfer pathways from pyruvate into downstream metabolites through glycolysis, pyruvate metabolism, and the tricarboxylic acid (TCA) cycle. Red circles denote ^13^C-labeled carbons originating from the tracer. Measured intracellular MIDs (blue) and predicted (orange) are shown.

To evaluate the mechanistic model’s ability to represent central carbon metabolism in iPSCs, simulated MIDs were compared with representative experimental labeling data obtained under the Historic Static HGLL condition using [1,2-^13^C_2_] glucose and [U-^13^C_5_] glutamine as tracers. After validating model performance against the Historic Static datasets, the model’s predictive capability was further assessed under the Static Pyruvate condition—without any parameter refitting—using [U-^13^C_3_] pyruvate as the isotopic tracer. The results are shown in Fig. 7. The model successfully captures the pyruvate-driven labeling patterns, including strong M+3 enrichment in pyruvate-derived metabolite pools and a corresponding reduction in contributions from glucose-derived labeling.

Predictive accuracy decreases for isotopomer distributions of downstream TCA cycle intermediates. This reduced performance likely reflects the simplified treatment of mitochondrial transport and compartmentalization, retention of labeled carbon within cytosolic pools, and the inherently low signal-to-noise ratios associated with TCA-cycle labeling measurements. These results suggest that incorporating more detailed representations of mitochondrial transport processes and compartment-specific metabolite pools may be necessary to improve quantitative prediction of TCA-cycle labeling in future model refinements.

#### 6.1.3 Model Prediction on Redox Dynamics

**Figure 8:**
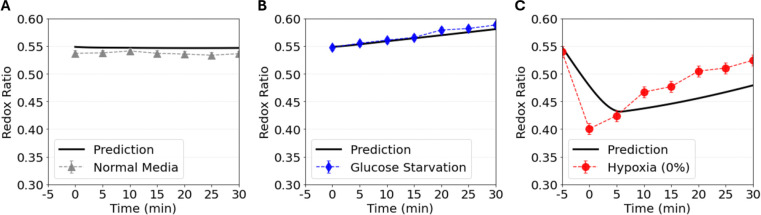
Predicted redox ratio dynamics under different environmental perturbations. (A) Normal medium, (B) glucose starvation, and (C) hypoxia (0% O_2_). Solid black lines denote model predictions for iPSC, while symbols represent experimental measurements. Data were reported in [31].

To further validate the single-cell metabolic model, we performed simulations to predict intracellular redox dynamics under different culture perturbations. Fig. 8 shows the simulated dynamics of the intracellular redox ratio, defined as NAD^+^/(NAD^+^ + NADH), across distinct culture conditions. Under normal medium conditions, the predicted redox ratio remains relatively stable throughout the simulation, reflecting sustained redox homeostasis (Fig. 8A). In this regime, NADH generated through glycolysis and the tricarboxylic acid (TCA) cycle is efficiently reoxidized to NAD^+^ via lactate dehydrogenase (LDH) activity and oxidative phosphorylation (OXPHOS), resulting in a balanced production and consumption of reducing equivalents.

Under glucose starvation, the model predicts a modest increase in the redox ratio (Fig. 8B). This behavior arises from diminished NADH generation due to reduced glycolytic flux, while mitochondrial oxidation of existing NADH continues. The transient imbalance between NADH production and consumption leads to a temporary elevation of the NAD^+^ fraction before a new steady state is reached.

In contrast, under severe hypoxia (0% O_2_), the redox ratio exhibits a sharp decline followed by a prolonged recovery period upon restoration of normoxic conditions (Fig. 8C). Suppression of OXPHOS under anoxic conditions limits mitochondrial NADH oxidation and NAD^+^ regeneration, leading to an accumulation of reduced cofactors, particularly NADH relative to NAD^+^. This shift toward a more reduced intracellular state reflects impaired electron transport chain activity and disruption of normal mitochondrial redox balance. The delayed recovery after oxygen restoration likely reflects the time required to reestablish mitochondrial oxidative capacity and resume normal tricarboxylic acid (TCA) cycle turnover, which depends on NAD^+^ regeneration to sustain oxidative metabolism [8, 9].

Overall, the simulated redox responses capture the qualitative trends expected from redox metabolic regulation under nutrient and oxygen perturbations. Validation against experimental measurements therefore focuses on reproducing the directionality and temporal patterns of redox changes rather than achieving direct quantitative agreement. The available experimental dataset [31] was obtained from a different cell line, which may exhibit distinct metabolic capacities and baseline NAD^+^/(NAD^+^ + NADH) ratios. In addition, the hypoxia induction protocol used in the referenced study likely altered medium pH due to carbon dioxide depletion during prolonged nitrogen bubbling, a procedural detail that was not explicitly reported. Such pH shifts can substantially influence enzyme kinetics and redox equilibria, thereby affecting measured redox ratios. As the iPS cell line was different, consequently, model validation emphasizes consistency in dynamic redox trends rather than absolute numerical agreement.

### 6.2 Aggregate Model Prediction Performance

### (1) Model prediction performance on aggregate size and distribution $\overset{ˆ}{\phi }(x,t)$.

**Figure 9:**
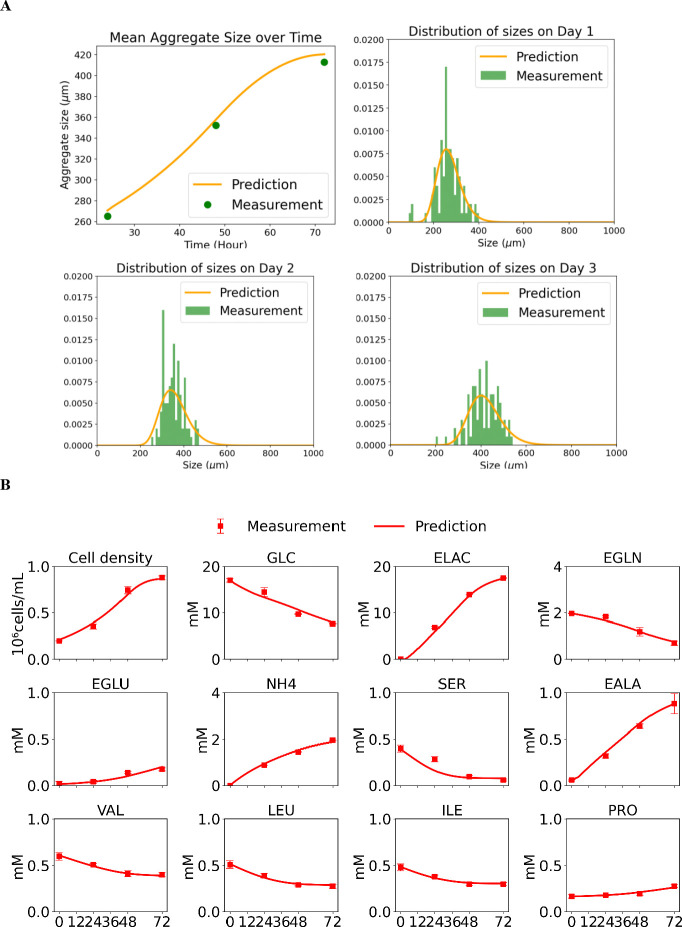
Aggregate growth, size distribution, and metabolic predictions compared with experimental measurements. **(A)** Predicted and measured mean iPSC aggregate size over time, together with aggregate size distributions on Days 1–3. Solid lines denote model predictions, data points represent experimental measurements with standard deviations. **(B)** Predicted and measured cell density and extracellular metabolite concentrations for aggregate cultures. Symbols indicate experimental measurements (mean ± standard deviation), and solid lines show model predictions. Metabolites include extracellular glucose (GLC), extracellular lactate (ELAC), extracellular glutamine (EGLN), extracellular glutamate (EGLU), extracellular ammonia (NH4), extracellular serine (SER), extracellular alanine (EALA), extracellular valine (VAL), extracellular leucine (LEU), extracellular isoleucine (ILE), and extracellular proline (PRO).

The model-predicted evolution of iPSC aggregate size over multiple culture days is shown in Fig. 9A. The model accurately captures both the mean aggregate size trajectory and the full size-distribution dynamics. Overall, the prediction curves align well with the experimental measurements, demonstrating strong performance in reproducing aggregate growth and distributional changes over time.

### (2) Model prediction performance for extracellular metabolites in aggregate cultures.

The model’s predictions for VCD, GLC, LAC, GLN, GLU, NH_4_, SER, ALA, VAL, LEU, ILE, and PRO are shown in Fig. 9B. In the figure, red dots represent experimental measurements from iPSC aggregate cultures, while red lines depict the model predictions. Overall, the model demonstrates strong predictive performance across all metabolites. Glucose and glutamine are steadily consumed, while lactate and ammonium accumulate, reflecting the characteristic metabolic behaviors of glycolysis, glutaminolysis, and nitrogen metabolism in proliferating iPSCs. Likewise, amino acids such as valine, leucine, and isoleucine show consistent depletion, indicating that the model effectively captures the dominant metabolic fluxes.

All prediction errors were calculated using the mean absolute percentage error (MAPE). The MAPE values for all metabolites are reported in Table 2. Overall, the predictions for most metabolites are accurate. The relatively larger error observed for extracellular serine (ESER) is likely attributable to the very low concentration range of this metabolite. In the aggregate cultures, ESER concentrations decrease to values close to 0.05–0.1 mM at later cell culture phase, where measurement noise and analytical uncertainty can significantly amplify percentage-based error metrics such as MAPE. The relatively higher prediction error for extracellular glutamate (EGLU) likely arises from the intricate regulation of glutamine–glutamate metabolism. As a central intermediate linking carbon and nitrogen metabolic pathways, glutamate levels are highly sensitive to subtle shifts in intracellular flux distributions [23]. Small deviations in pathways such as glutaminolysis, transamination, or TCA cycle anaplerosis can therefore lead to amplified changes in extracellular glutamate. Moreover, spatial heterogeneity within aggregates can further modulate local glutamine uptake and glutamate secretion, introducing additional variability that is challenging for a single-cell model to capture.

## 7 Discussion

### (1) Multi-Scale Foundational Modeling and Experimental Validation.

This study introduces a multiscale foundational modeling framework that characterizes iPSC manufacturing across a broad spectrum of culture configurations—from the relatively homogeneous monolayer systems commonly used in laboratory settings to the heterogeneous aggregate cultures favored for large-scale industrial production. At the core of the framework is an expanded single-cell mechanistic metabolic model that integrates extracellular culture dynamics with intracellular metabolic flux distributions and cellular redox states. To represent aggregate cultures, the framework incorporates additional multiscale modules that capture cell–cell interactions and spatial heterogeneity. A population balance model describes aggregate formation and size-distribution dynamics arising from cell aggregation and proliferation, while a reaction–diffusion model resolves nutrient transport and metabolic gradients within aggregates. Together, these modular components allow the multiscale framework to flexibly represent both monolayer and aggregate systems with diverse forms of heterogeneity.

To construct and validate the framework, systematic monolayer and aggregate culture experiments were performed. The resulting datasets include cell growth measurements, extracellular metabolite profiles, aggregate size dynamics, mass isotopomer distributions (MIDs) obtained from isotope tracer experiments, and non-destructive optical redox measurements. These complementary datasets provide comprehensive information for model calibration and validation across the Historic Static, Static Pyruvate, and Aggregate culture conditions. By integrating these multi-modal measurements, the framework enables quantitative evaluation of metabolic behaviors across distinct culture configurations and supports predictive modeling of iPSC metabolism under heterogeneous manufacturing environments.

### (2) Monolayer Cultures and Single-cell Mechanistic Modeling.

The relative homogeneity of monolayer cultures offers a well-controlled setting for investigating single-cell metabolic responses to specific extracellular perturbations. In these systems, cells experience nearly uniform concentrations of nutrients, oxygen, and metabolites, minimizing spatial heterogeneity and reducing confounding effects from transport limitations. As a result, intracellular metabolic regulation can be interpreted more directly, making monolayer cultures an ideal setting for elucidating intrinsic single-cell metabolic behavior. These insights form the foundational building block of the broader multiscale mechanistic modeling framework.

Because ^13^C metabolic flux analysis (^13^C-MFA) uniquely enables quantitative estimation of intracellular reaction rates, multiple isotope-labeling experiments were employed to probe metabolic responses to environmental perturbations. Building on our previous ^13^C-MFA studies of iPSC metabolism in static monolayer cultures [22], a set of complementary isotopic tracers was used to interrogate distinct segments of central carbon metabolism. Specifically, [U-^13^C_6_]glucose was applied to trace glycolytic flux and carbon entry into the TCA cycle through both pyruvate dehydrogenase (PDH) and pyruvate carboxylase (PC) pathways. [U-^13^C_5_]glutamine was used to characterize glutaminolysis and anaplerotic carbon input into the TCA cycle through *α*-ketoglutarate. In addition, [1,2-^13^C_2_]glucose was used to differentiate glycolytic flux from pentose phosphate pathway (PPP) activity by generating distinct isotopomer labeling patterns in downstream metabolites such as lactate and alanine. Together, these tracers provide comprehensive coverage of carbon flux through glycolysis, the TCA cycle, anaplerotic reactions, and the pentose phosphate pathway.

In this study, an additional tracer, [U-^13^C_3_]pyruvate, was introduced to further resolve carbon flow through pyruvate metabolism and the tricarboxylic acid (TCA) cycle. Experimental observations showed that supplementation with extracellular pyruvate significantly alters central carbon metabolism. Under Static Pyruvate conditions, glucose consumption decreased and the glutamine-to-glutamate conversion ratio shifted, indicating partial substrate substitution and feedback regulation of glycolytic and anaplerotic pathways. Mass isotopomer distribution (MID) analysis (Fig. 7) confirmed incorporation of [U-^13^C_3_]pyruvate into downstream metabolites, demonstrating its participation in central carbon metabolism and constraining its partitioning between cytosolic and mitochondrial pathways.

### (3) Extension to Heterogeneous Aggregate Cultures through Multiscale Modeling.

Building on the mechanistic understanding established at the single-cell level, the multi-scale modeling framework was extended to aggregate cultures by incorporating additional modules describing aggregation dynamics and intra-aggregate transport processes. In contrast to monolayer cultures, aggregate systems develop heterogeneous microenvironments due to diffusion limitations and extensive cell–cell interactions. Resulting gradients in oxygen, nutrients, and metabolic byproducts create spatial variability in cellular metabolism that cannot be captured by single-cell models alone.

Experimental observations in aggregate cultures revealed coordinated metabolic adaptations driven by these heterogeneous conditions. Compared with monolayer cultures, aggregates exhibited reduced glucose consumption and lactate production, consistent with oxygen and nutrient gradients that reshape central carbon metabolism. Diffusion-limited oxygen availability in the aggregate interior likely suppresses oxidative metabolism and alters the distribution of glycolytic flux. Reduced glutamine consumption and glutamate production further indicate a reorganization of nitrogen metabolism in response to spatial heterogeneity. Notably, ammonia production remained comparable across culture formats, suggesting compensatory interactions among glutamine uptake, transamination reactions, and downstream nitrogen processing. Collectively, these results suggest that metabolic changes in aggregates arise from redistribution of metabolic fluxes rather than uniform downregulation of metabolic activity.

Importantly, both monolayer and aggregate cultures can be represented within the unified multiscale foundational modeling framework, enabling integration of heterogeneous observations across diverse manufacturing systems and facilitating scale-up. In monolayer cultures, homogeneous extracellular conditions allow the framework to resolve intrinsic single-cell metabolic regulation. In aggregate cultures, additional modules describing aggregation dynamics and reaction–diffusion transport capture the heterogeneous microenvironment that shapes cellular metabolism at larger manufacturing scales. This unified representation enables the framework to flexibly describe iPSC culture behavior across manufacturing configurations, from controlled laboratory monolayer systems to heterogeneous aggregate cultures used in large-scale bioproduction.

Collectively, this work demonstrates the value of integrating isotopic labeling, extracellular metabolite measurements, optical redox sensing, and multiscale transport modeling within a unified modeling and analytical framework. Establishing intracellular flux-regulation mechanisms under controlled static conditions enables mechanistic interpretation of the metabolic reorganization that emerges in aggregate cultures when spatial heterogeneity is introduced. From an engineering perspective, this integrated approach provides a foundation for predictive modeling strategies that can inform monitoring, optimization, and control of scalable iPSC bioprocesses.

### (4) Limitations and Future Research.

While the present framework focuses on a defined reaction network and selected regulatory mechanisms, several extensions could further enhance its predictive capability. Incorporating compartment-specific transport processes, more detailed representations of mitochondrial dynamics, and additional layers of transcriptional regulation may improve quantitative accuracy, particularly under dynamic or perturbed conditions. Furthermore, coupling the modeling framework with real-time sensing and control strategies represents a promising direction toward closed-loop optimization of aggregate culture systems and the robust, scalable design of iPSC manufacturing processes.

## Supplementary Material

Supplement 1

## Figures and Tables

**Table 1:** Experimental conditions for the Historic Static [22], Static Pyruvate, and Aggregate cultures.

Experiment	Historic Static Cultures	Static Pyruvate Cultures	Aggregate Cultures

**Media Composition**			
Initial glucose (mM) (LG/HG)	5.6 / 18.3	10.4 / 33.6	17.0
Initial glutamine (mM)	2.7	4.69	1.84
Initial lactate (mM) (LL/HL)	0 / 20	0 / 38	0
Initial pyruvate (mM)	0.41	4.4	0
Initial cell density or equivalent (cells/mL)	3.2×10^4^	4.8×10^4^	2.0×10^5^
Initial cell density (cells/cm^2^)	~ 1×10^4^	~ 1×10^4^	N/A
Initial pH	N/A	N/A	7.5
Final pH	N/A	N/A	6.5
Labeled substrates	[1,2-^13^C_2_] glucose [U-^13^C_5_] glutamine [U-^13^C_3_] lactate	[U-^13^C_3_] pyruvate	No
Other additions	20 mM NaCl (HGLL, LGLL)	20 mM NaCl (HGLL, LGLL)	N/A

**Culture Setup**			
Culture method	6-well plates; 100-mm Petri dishes	6-well plates; T75 flasks	Shake flasks (125 mL)
Working volume (mL)	3	2	22–28

**Environmental Conditions**			
Temperature (°C)	37	37	37
CO_2_ concentration (%)	5	5	5 70 (D0–D1)
Agitation rate (rpm)	N/A	N/A	75 (D2–D4) 80 (D5)
Shake flask type	N/A	N/A	Baffled, PC

**Process Parameters**			
Passage number	P3	P3	P3
Culture duration (days)	2	2	5
Feeding (day)	N/A	N/A	3

**Data Collection**			
VCD method	Vi-Cell XR (Beckman)	Vi-Cell XR (Beckman)	Nova Flex 2
VCD replicates	6	2	3
Metabolites	Cedex Bio	Cedex Bio	Nova Flex 2
Metabolite replicates	6	2	3
Amino acid analysis method	EZ:faast kit (Phenomenex)	REBEL (908 Devices)	REBEL (908 Devices)
AA replicates (Tech/Bio)	1 / 6	2 / 2	2 / 3
Redox kit replicates	N/A	N/A	1
Redox TPE	N/A	N/A	2 mL in 50 mL conical tube

**Abbreviations:** Tech = technical (repeated-measures) replicates; Bio = biological replicates; HG = high glucose; LG = low glucose; HL = high lactate; LL = low lactate; D = day of culture; PC = polycarbonate; P = passage number.

**Table 2:** Mean absolute percentage error (MAPE) for all metabolites.

Compound	MAPE (%)	Compound	MAPE (%)

EGLC	7.02	ELAC	5.21
EGLN	6.02	EGLU	32.73
NH_4_	5.36	ESER	29.00
EALA	5.69	EVAL	2.11
ELEU	4.56	EILE	3.22
EPRO	4.69		

## Data Availability

Data will be made available on request.
