# Supplementary material for: Intracellular Measurement-Informed Multiscale Modeling for Scalable iPSC Manufacturing
Source: ArXiv. 2026 Mar 17:arXiv:2603.17090v1. Preprint. [Version 1] (PMC13019451)
Supplement: Supplement 1 [file NIHPP2603.17090v1-supplement-1.pdf]

Appendix. Supporting Information

Table S1: Abbreviations for intracellular metabolites used in the MID analysis.

Abbreviation	Metabolite name	Abbreviation	Metabolite name
GLC	Glucose	3PG	3-Phosphoglycerate
PEP	Phosphoenolpyruvate	PYR	Pyruvate
LAC	Lactate	ALA	Alanine
CIT	Citrate	AKG	α-Ketoglutarate
ACO	Aconitate	ASP	Aspartate
ASN	Asparagine	CYS	Cysteine
VAL	Valine	PHE	Phenylalanine
TRP	Tryptophan	HIS	Histidine

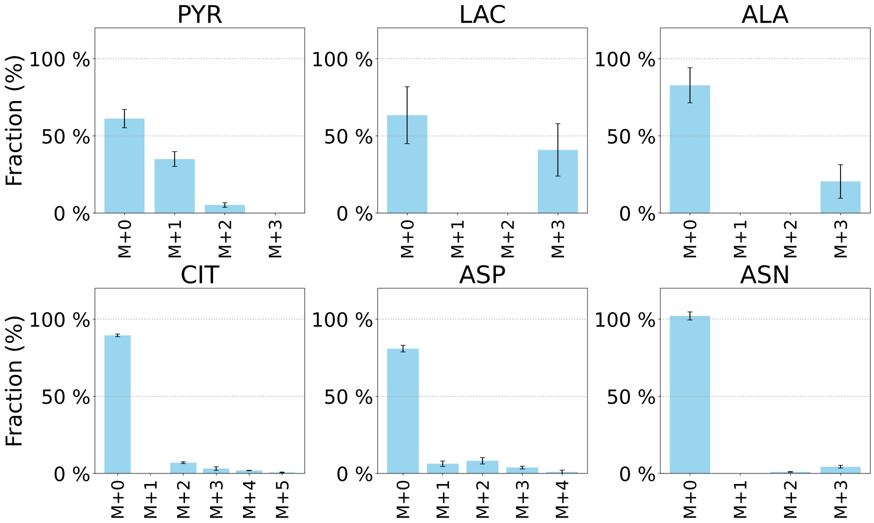

Figure S2: Intracellular Mass isotopomer distribution (MID) data for the Static Pyruvate LGHL culture condition at 48 h. MID data were corrected for natural isotopic abundance. Standard abbreviations are used for metabolites.

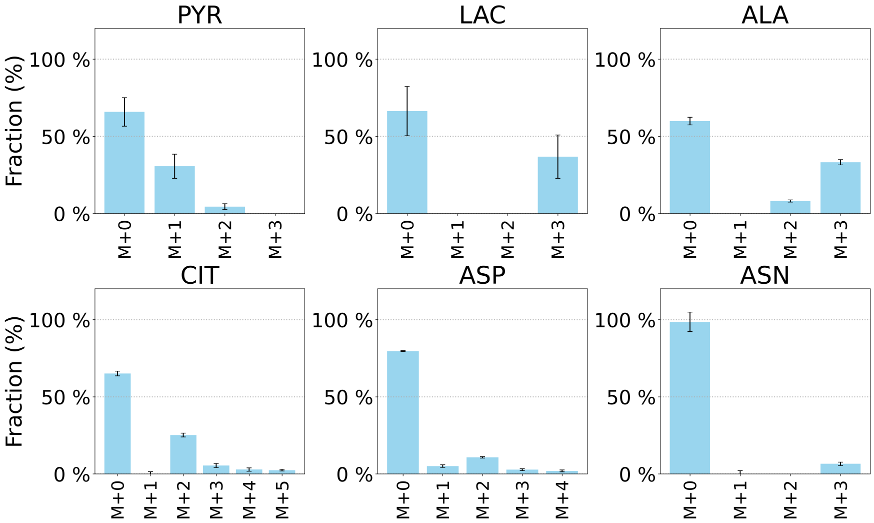

Figure S3: Intracellular Mass isotopomer distribution (MID) data for the Static Pyruvate LGLL culture condition at 48 h. MID data were corrected for natural isotopic abundance. Standard abbreviations are used for metabolites.

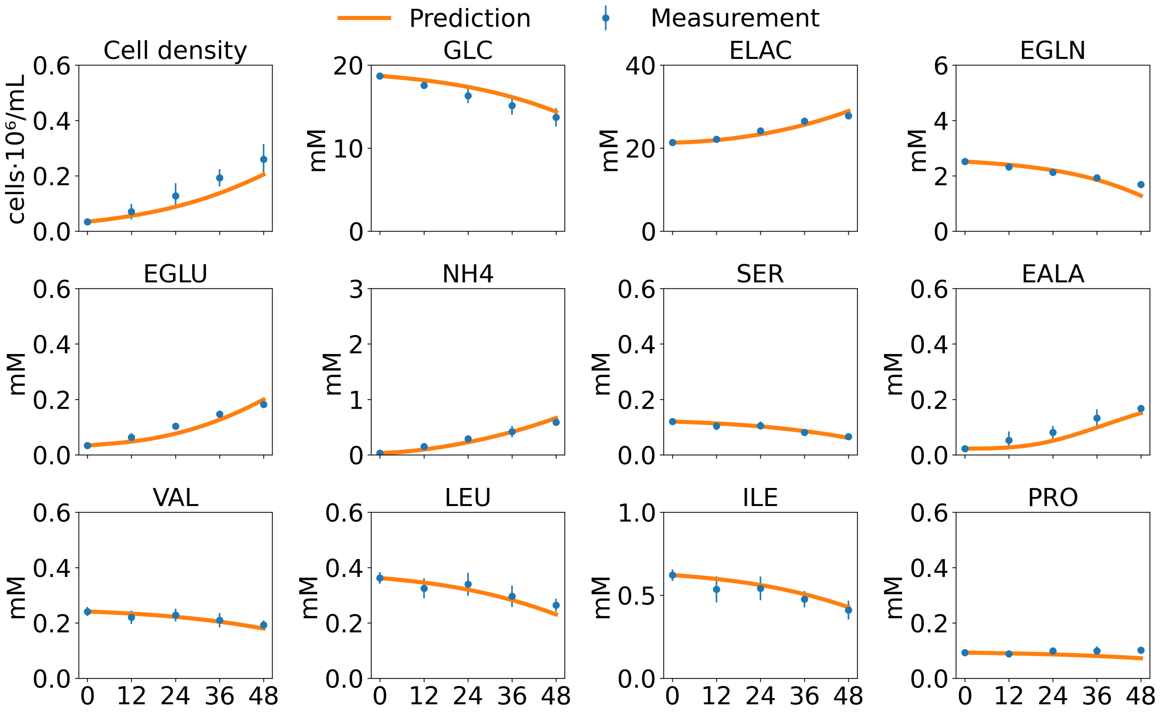

Figure S4: Dynamic model cross validation for HGHL condition left with model trained on three other Historic Static culture datasets and all Static Pyruvate culture datasets.

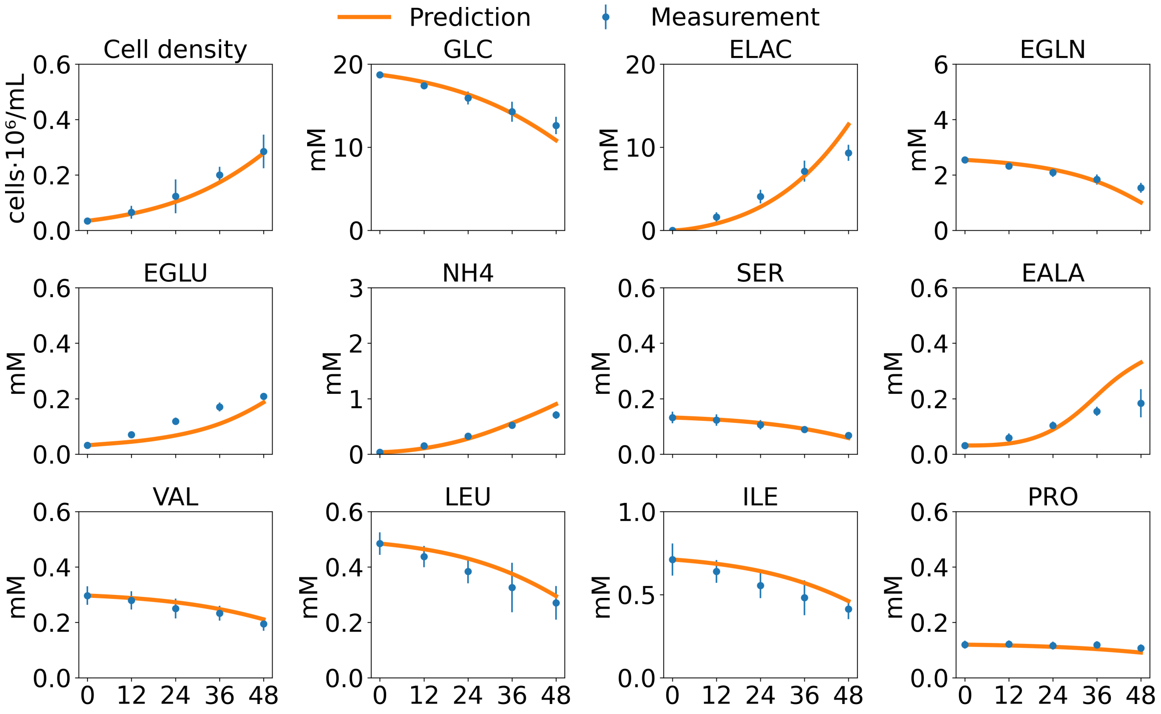

Figure S5: Dynamic model cross validation for HGLL condition left with model trained on three other Historic Static culture datasets and all Static Pyruvate culture datasets.

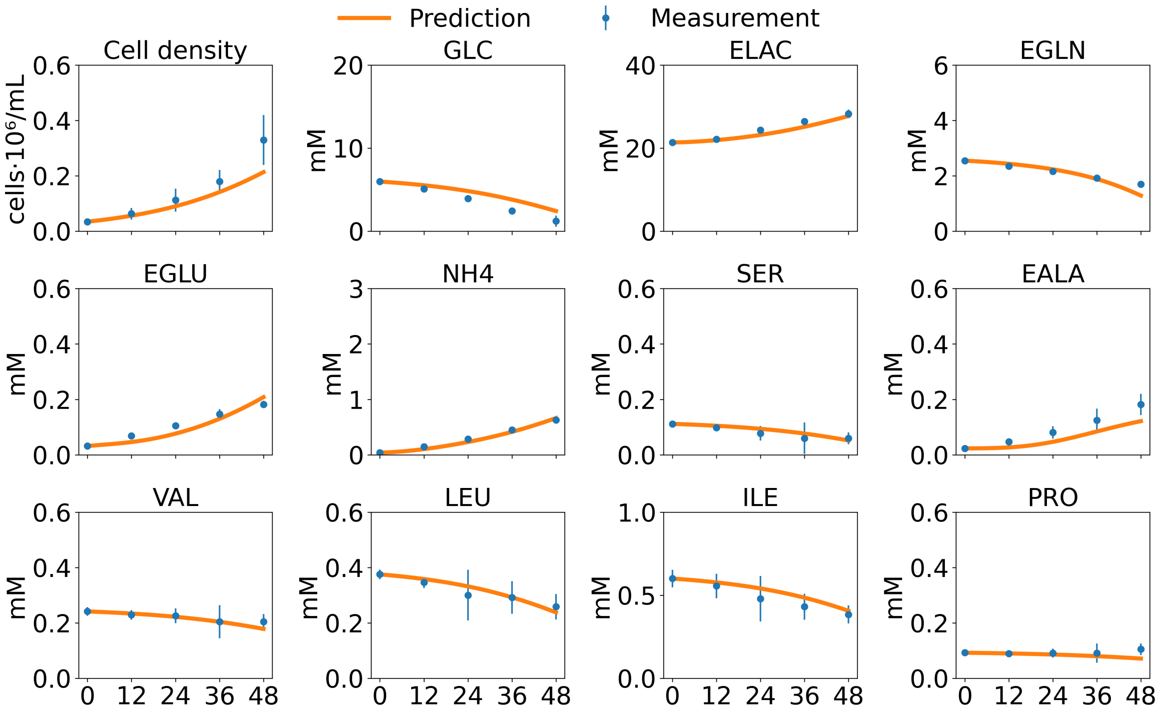

Figure S6: Dynamic model cross validation for LGHL condition left with model trained on three other Historic Static culture datasets and all Static Pyruvate culture datasets.

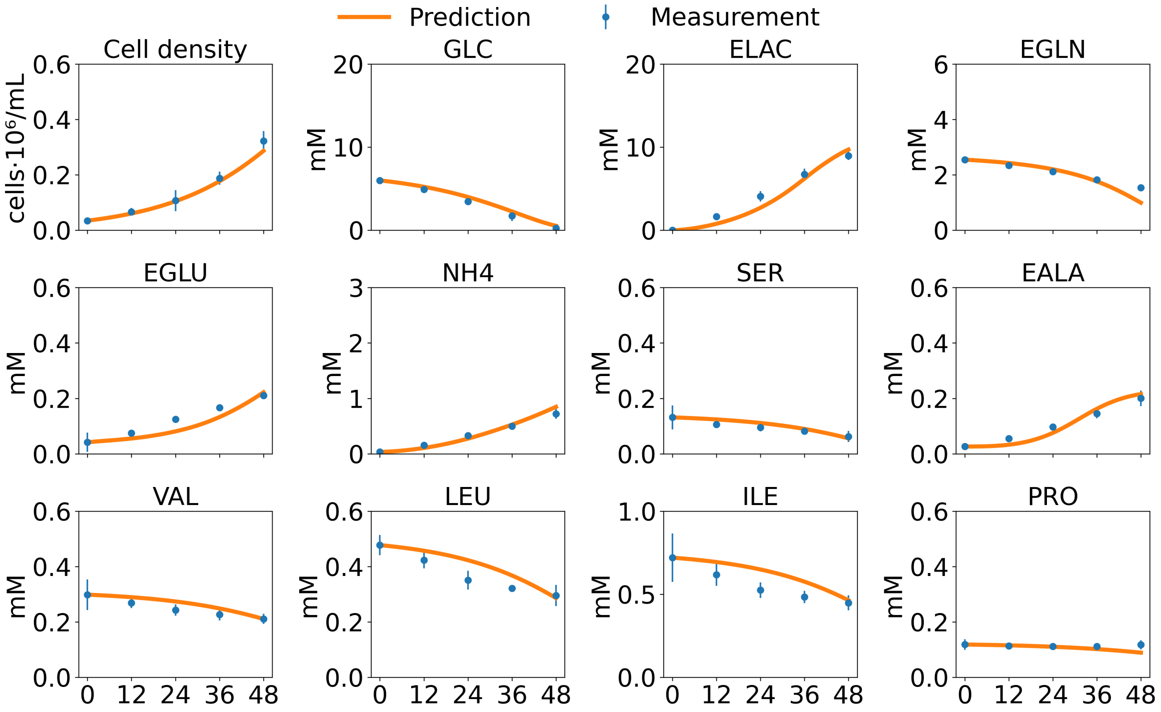

Figure S7: Dynamic model cross validation for LGLL condition left with model trained on three other Historic Static culture datasets and all Static Pyruvate culture datasets.

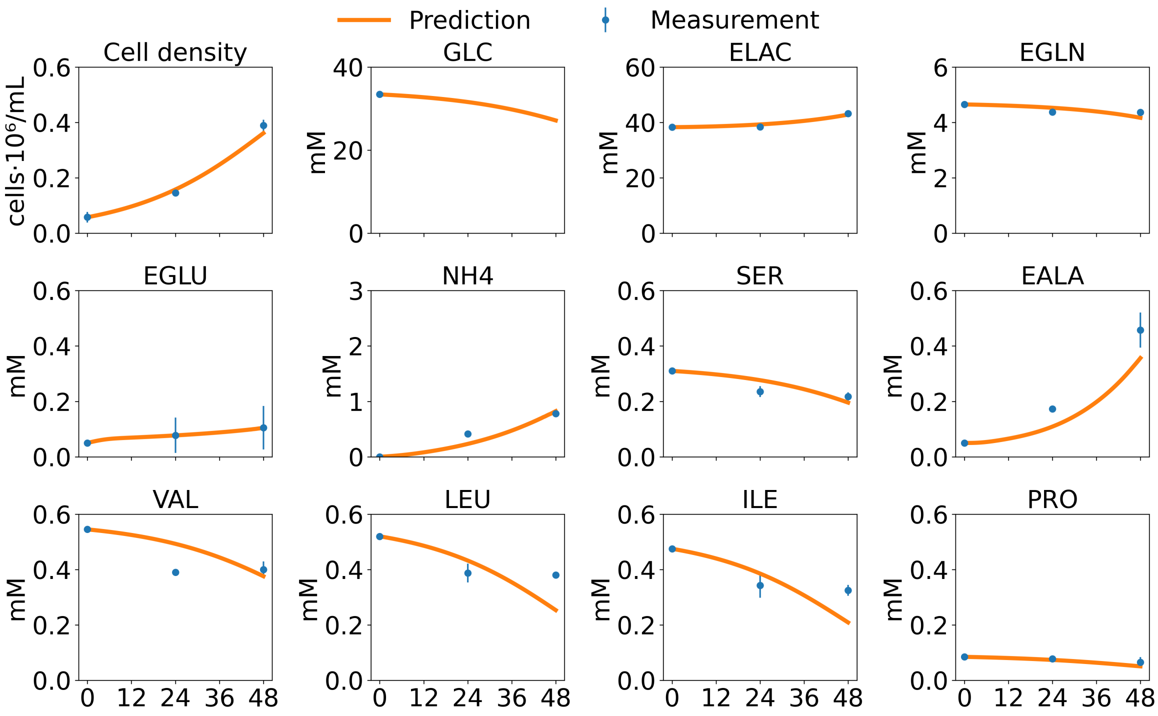

Figure S8: Dynamic model cross validation for HGHL condition left with model trained on three other Static Pyruvate Culture datasets and all Historic Static Culture datasets.

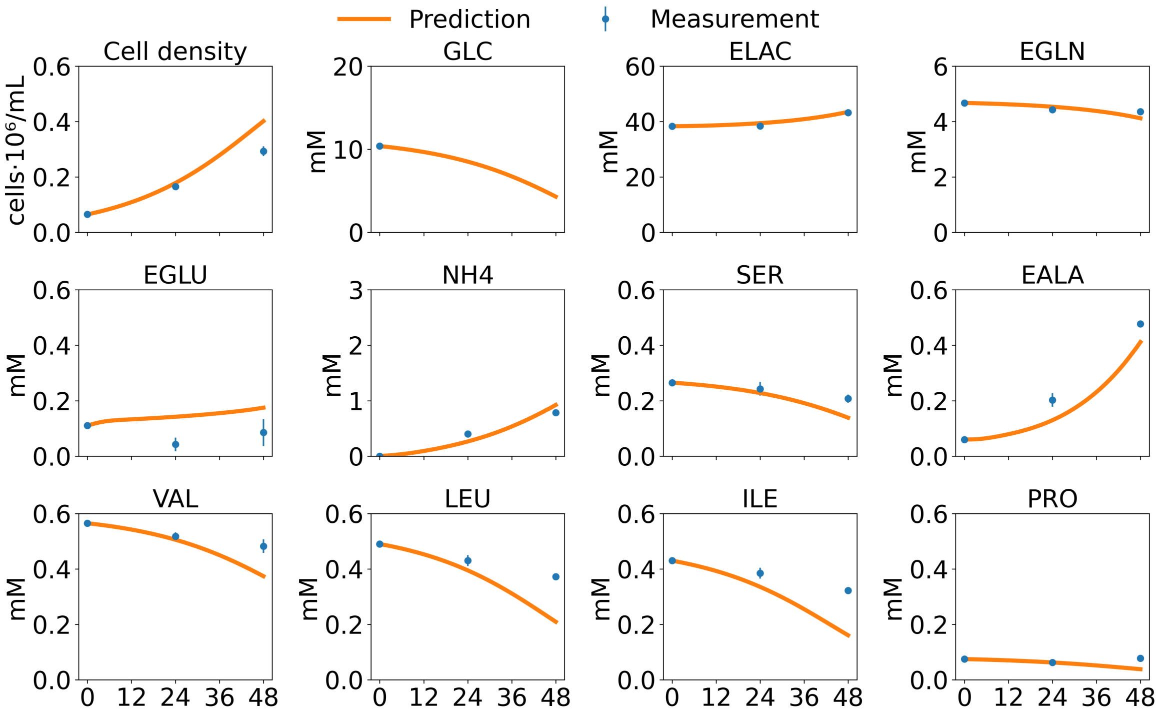

Figure S9: Dynamic model cross validation for LGHL condition left with model trained on three other Static Pyruvate Culture datasets and all Historic Static Culture datasets.

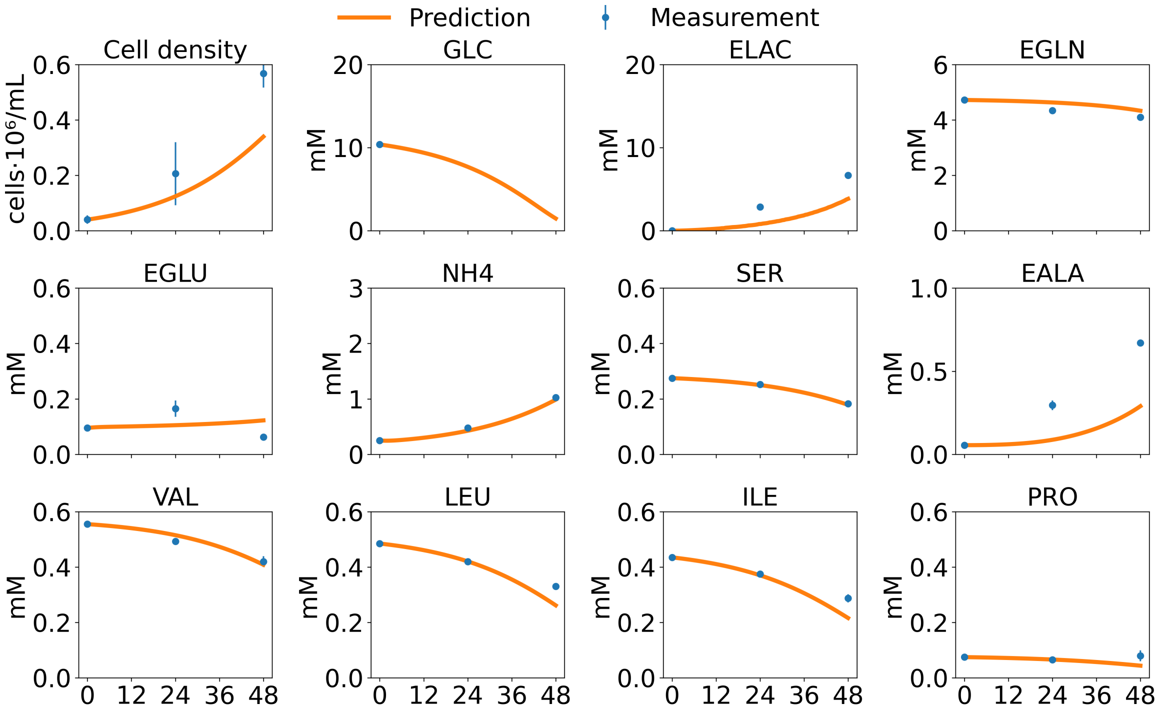

Figure S10: Dynamic model cross validation for LGLL condition left with model trained on three other Static Pyruvate Culture datasets and all Historic Static Culture datasets.

Table S2: Reaction network for single-cell model including the carbon transitions.

Pathway	Reaction
**Glycolysis**	
V_1	$\mathrm{GLC}(abcdef) + 2\mathrm{NAD}^+ \rightarrow 2\mathrm{PYR}(cba) + \mathrm{PYR}(def) + 2\mathrm{NADH}$
V_2	$\mathrm{PYR}(abc) + \mathrm{NADH} \rightleftharpoons \mathrm{LAC}(abc) + \mathrm{NAD}^+$
**TCA**	
V_3	$\mathrm{PYR}(abc) + \mathrm{NAD}^+ \rightarrow \mathrm{ACCOA}(bc) + \mathrm{NADH} + \mathrm{CO_2}(a)$
V_4	$\mathrm{PYR}(abc) + \mathrm{CO_2}(d) \rightarrow \mathrm{OAA}(abcd)$
V_5	$\mathrm{ACCOA}(ab) + \mathrm{OAA}(cdef) \rightarrow \mathrm{CIT}(fedbac)$
V_6	$\mathrm{CIT}(abcdef) + \mathrm{NAD}^+ \rightarrow \mathrm{AKG}(abcde) + \mathrm{NADH} + \mathrm{CO_2}(f)$
V_7	$\mathrm{AKG}(abcde) + \mathrm{COA} + \mathrm{NAD}^+ \rightarrow \mathrm{SUCCOA}(abcd) + \mathrm{NADH} + \mathrm{CO_2}(e)$
V_8	$\mathrm{SUCCOA}(abcd) + 2/3\mathrm{NAD}^+ \rightarrow \mathrm{FUM}(abcd) + 2/3\mathrm{NADH}$
V_9	$\mathrm{FUM}(abcd) \rightarrow \mathrm{MAL}(abcd)$
V_{10}	$\mathrm{MAL}(abcd) + \mathrm{NAD}^+ \rightarrow \mathrm{OAA}(abcd) + \mathrm{NADH}$
V_{11}	$\mathrm{MAL}(abcd) + \mathrm{NADP}^+ \rightarrow \mathrm{PYR}(abc) + \mathrm{NADPH} + \mathrm{CO_2}(d)$
**AA**	
V_{12}	$\mathrm{GLN}(abcde) \rightleftharpoons \mathrm{GLU}(abcde) + \mathrm{NH_4}$
V_{130}	$\mathrm{PRO}(abcde) + \mathrm{NAD}^+ \rightarrow \mathrm{GLU}(abcde) + \mathrm{NADH}$
V_{14}	$\mathrm{HIS}(abcdef) \rightarrow \mathrm{GLU}(abcde) + \mathrm{NH_4} + \mathrm{THF}(f)$
V_{15}	$\mathrm{GLU}(abcde) + \mathrm{NAD}^+ \rightarrow \mathrm{AKG}(abcde) + \mathrm{NADH} + \mathrm{NH_4}$
V_{16}	$\mathrm{GLU}(abcde) + \mathrm{PYR}(fgh) \rightleftharpoons \mathrm{AKG}(abcde) + \mathrm{ALA}(fgh)$
V_{17}	$\mathrm{SER}(abc) \rightarrow \mathrm{PYR}(abc) + \mathrm{NH_4}$
V_{18}	$\mathrm{ASN}(abcd) \rightarrow \mathrm{ASP}(abcd) + \mathrm{NH_4}$
V_{19}	$\mathrm{ASP}(abcd) + \mathrm{AKG}(efghi) \rightleftharpoons \mathrm{GLU}(efghi) + \mathrm{OAA}(abcd) + \mathrm{NH_4}$
V_{20}	$\mathrm{ILE}(abcde) + \mathrm{CO_2}(f) + 2\mathrm{COA} \rightarrow \mathrm{ACCOA}(ab) + \mathrm{SUCCOA}(fcde)$
**Oxidative Phosphoryla-tion**	
V_{21}	$\mathrm{O_2} + 2\mathrm{NADH} \rightarrow 2\mathrm{NAD}^+ + 2\mathrm{H_2O}$
**Transport**	
V_{22}	$\mathrm{EPYR}(abc) \rightarrow \mathrm{PYR}(abc)$
V_{23}	$\mathrm{ALA}(abc) \rightarrow \mathrm{EALA}(abc)$
V_{24}	$\mathrm{GLU}(abcde) \rightarrow \mathrm{EGLU}(abcde)$
V_{25}	$\mathrm{EGLN}(abcde) \rightarrow \mathrm{GLN}(abcde)$
V_{26}	$\mathrm{EASP}(abcd) \rightarrow \mathrm{ASP}(abcd)$
V_{27}	$\mathrm{LAC}(abc) \rightarrow \mathrm{ELAC}(abc)$
V_{28}	$\mathrm{O}_{2(aq)} \rightarrow \mathrm{O_2}$
**Biomass**	
V_{29}	$0.16\mathrm{GLC} + 0.1\mathrm{GLN} + 0.151\mathrm{GLU} + 0.044\mathrm{ALA} + 0.023\mathrm{HIS} + 0.047\mathrm{ILE} + 0.088\mathrm{LEU} + 0.085\mathrm{LYS} + 0.04\mathrm{SER} + 0.033\mathrm{TYR} + 0.05\mathrm{VAL} + 0.039\mathrm{THR} + 0.065\mathrm{PRO} + 0.047\mathrm{PHE} + 0.011\mathrm{MET} \rightarrow X$

Table S3: Flux rate models for the single-cell.

#	**Flux equation**
1	$v(\mathrm{HK}) = v_{\max\ \mathrm{HK}} \cdot \frac{\mathrm{GLC}}{K_{m\mathrm{GLC}}+\mathrm{GLC}} \cdot \frac{K_{i\mathrm{LACtoHK}}}{K_{i\mathrm{LACtoHK}}+\mathrm{LAC}}$
2	$v(\mathrm{LDH}) = v_{\mathrm{maxf\ LDH}} \cdot \frac{\mathrm{PYR}}{K_{m\mathrm{PYR}}+\mathrm{PYR}} \cdot \frac{\frac{\mathrm{NADH}}{\mathrm{NAD}}}{K_{m\frac{\mathrm{NADH}}{\mathrm{NAD}}}+\frac{\mathrm{NADH}}{\mathrm{NAD}}} - v_{\mathrm{maxr\ LDH}} \cdot \frac{\mathrm{LAC}}{K_{m\mathrm{LAC}}+\mathrm{LAC}} \cdot \frac{\frac{\mathrm{NAD}}{\mathrm{NADH}}}{K_{m\frac{\mathrm{NAD}}{\mathrm{NADH}}}+\frac{\mathrm{NAD}}{\mathrm{NADH}}}$
3	$v(\mathrm{PDH}) = v_{\max\ \mathrm{PDH}} \cdot \frac{\mathrm{PYR}}{K_{m\mathrm{PYR}}+\mathrm{PYR}} \cdot \frac{\frac{\mathrm{NAD}}{\mathrm{NADH}}}{K_{m\frac{\mathrm{NAD}}{\mathrm{NADH}}}+\frac{\mathrm{NAD}}{\mathrm{NADH}}}$
4	$v(\mathrm{PC}) = v_{\max\ \mathrm{PC}} \cdot \frac{\mathrm{PYR}}{K_{m\mathrm{PYR}}+\mathrm{PYR}}$
5	$v(\mathrm{CS}) = v_{\max\ \mathrm{CS}} \cdot \frac{\mathrm{ACCOA}}{K_{m\mathrm{ACCOA}}+\mathrm{ACCOA}} \cdot \frac{\mathrm{OAA}}{K_{m\mathrm{OXA}}+\mathrm{OAA}}$
6	$v(\mathrm{CITS/ISOD}) = v_{\max\ \mathrm{CITS/ISOD}} \cdot \frac{\mathrm{CIT}}{K_{m\mathrm{CIT}}+\mathrm{CIT}} \cdot \frac{\frac{\mathrm{NAD}}{\mathrm{NADH}}}{K_{m\frac{\mathrm{NAD}}{\mathrm{NADH}}}+\frac{\mathrm{NAD}}{\mathrm{NADH}}}$
7	$v(\mathrm{AKGDH}) = v_{\max\ \mathrm{AKGDH}} \cdot \frac{\mathrm{AKG}}{K_{m\mathrm{AKG}}+\mathrm{AKG}} \cdot \frac{\frac{\mathrm{NAD}}{\mathrm{NADH}}}{K_{m\frac{\mathrm{NAD}}{\mathrm{NADH}}}+\frac{\mathrm{NAD}}{\mathrm{NADH}}}$
8	$v(\mathrm{SDH}) = v_{\max\ \mathrm{SDH}} \cdot \frac{\mathrm{SUCCOA}}{K_{m\mathrm{SUCCOA}}+\mathrm{SUCCOA}} \cdot \frac{\frac{\mathrm{NAD}}{\mathrm{NADH}}}{K_{m\frac{\mathrm{NAD}}{\mathrm{NADH}}}+\frac{\mathrm{NAD}}{\mathrm{NADH}}}$
9	$v(\mathrm{FUM}) = v_{\max\ \mathrm{FUM}} \cdot \frac{\mathrm{FUM}}{K_{m\mathrm{FUM}}+\mathrm{FUM}}$
10	$v(\mathrm{MDH}) = v_{\max\ \mathrm{MDH}} \cdot \frac{\mathrm{MAL}}{K_{m\mathrm{MAL}}+\mathrm{MAL}} \cdot \frac{\frac{\mathrm{NAD}}{\mathrm{NADH}}}{K_{m\frac{\mathrm{NAD}}{\mathrm{NADH}}}+\frac{\mathrm{NAD}}{\mathrm{NADH}}}$
11	$v(\mathrm{ME}) = v_{\max\ \mathrm{ME}} \cdot \frac{\mathrm{MAL}}{K_{m\mathrm{MAL}}+\mathrm{MAL}} \cdot \frac{\frac{\mathrm{NADP}}{\mathrm{NADPH}}}{K_{m\frac{\mathrm{NADP}}{\mathrm{NADPH}}}+\frac{\mathrm{NADP}}{\mathrm{NADPH}}}$
12	$v(\mathrm{GLNS}) = v_{\mathrm{maxf\ GLNS}} \cdot \frac{\mathrm{GLN}}{K_{m\mathrm{GLN}}+\mathrm{GLN}} - v_{\mathrm{maxr\ GLNS}} \cdot \frac{\mathrm{GLU}}{K_{m\mathrm{GLU}}+\mathrm{GLU}} \cdot \frac{\mathrm{NH_4}}{K_{m\mathrm{NH_4}}+\mathrm{NH_4}}$
13	$v(\mathrm{PRO}) = v_{\max\ \mathrm{PRO}} \cdot \frac{\mathrm{PRO}}{K_{m\mathrm{PRO}}+\mathrm{PRO}} \cdot \frac{\frac{\mathrm{NAD}}{\mathrm{NADH}}}{K_{m\frac{\mathrm{NAD}}{\mathrm{NADH}}}+\frac{\mathrm{NAD}}{\mathrm{NADH}}}$
14	$v(\mathrm{HIS}) = v_{\max\ \mathrm{HIS}} \cdot \frac{\mathrm{HIS}}{K_{m\mathrm{HIS}}+\mathrm{HIS}} \cdot \frac{\mathrm{AKG}}{K_{m\mathrm{AKG}}+\mathrm{AKG}}$
15	$v(\mathrm{GLDH}) = v_{\mathrm{maxf\ GLDH}} \cdot \frac{\mathrm{GLU}}{K_{m\mathrm{GLU}}+\mathrm{GLU}} \cdot \frac{\frac{\mathrm{NAD}}{\mathrm{NADH}}}{K_{m\frac{\mathrm{NAD}}{\mathrm{NADH}}}+\frac{\mathrm{NAD}}{\mathrm{NADH}}} - v_{\mathrm{maxr\ GLDH}} \cdot \frac{\mathrm{AKG}}{K_{m\mathrm{AKG}}+\mathrm{AKG}} \cdot \frac{\frac{\mathrm{NADH}}{\mathrm{NAD}}}{K_{m\frac{\mathrm{NADH}}{\mathrm{NAD}}}+\frac{\mathrm{NADH}}{\mathrm{NAD}}} \cdot \frac{\mathrm{NH_4}}{K_{m\mathrm{NH_4}}+\mathrm{NH_4}}$
16	$v(\mathrm{AlaTA}) = v_{\mathrm{maxf\ AlaTA}} \cdot \frac{\mathrm{GLU}}{K_{m\mathrm{GLU}}+\mathrm{GLU}} \cdot \frac{\mathrm{PYR}}{K_{m\mathrm{PYR}}+\mathrm{PYR}} - v_{\mathrm{maxr\ AlaTA}} \cdot \frac{\mathrm{ALA}}{K_{m\mathrm{ALA}}+\mathrm{ALA}} \cdot \frac{\mathrm{AKG}}{K_{m\mathrm{AKG}}+\mathrm{AKG}}$
17	$v(\mathrm{SAL}) = v_{\max\ \mathrm{SAL}} \cdot \frac{\mathrm{SER}}{K_{m\mathrm{SER}}+\mathrm{SER}}$
18	$v(\mathrm{ASN}) = v_{\max\ \mathrm{ASN}} \cdot \frac{\mathrm{ASN}}{K_{m\mathrm{ASN}}+\mathrm{ASN}}$
19	$v(\mathrm{ASTA}) = v_{\mathrm{maxf\ ASTA}} \cdot \frac{\mathrm{ASP}}{K_{m\mathrm{ASP}}+\mathrm{ASP}} \cdot \frac{\mathrm{NH_4}}{K_{m\mathrm{NH_4}}+\mathrm{NH_4}} - v_{\mathrm{maxr\ ASTA}} \cdot \frac{\mathrm{AKG}}{K_{m\mathrm{AKG}}+\mathrm{AKG}} \cdot \frac{\mathrm{GLU}}{K_{m\mathrm{GLU}}+\mathrm{GLU}} \cdot \frac{\mathrm{OAA}}{K_{m\mathrm{OXA}}+\mathrm{OAA}}$
20	$v(\mathrm{ILE}) = v_{\max\ \mathrm{ILE}} \cdot \frac{\mathrm{ILE}}{K_{m\mathrm{ILE}}+\mathrm{ILE}} \cdot \frac{\frac{\mathrm{NAD}}{\mathrm{NADH}}}{K_{m\frac{\mathrm{NAD}}{\mathrm{NADH}}}+\frac{\mathrm{NAD}}{\mathrm{NADH}}}$
21	$v(\mathrm{resp}) = v_{\max\ \mathrm{resp}} \cdot \frac{\mathrm{O_2}}{K_{m\mathrm{O_2}}+\mathrm{O_2}} \cdot \frac{\mathrm{NADH}}{K_{m\mathrm{NADH}}+\mathrm{NADH}}$
22	$v(\mathrm{PYRT}) = v_{\max\ \mathrm{PYRT}} \cdot \frac{\mathrm{EPYR}}{K_{m\mathrm{EPYR}}+\mathrm{EPYR}}$
23	$v(\mathrm{ALAT}) = v_{\max\ \mathrm{ALAT}} \cdot \frac{\mathrm{ALA}}{K_{m\mathrm{ALA}}+\mathrm{ALA}}$
24	$v(\mathrm{GLUT}) = v_{\max\ \mathrm{GLUT}} \cdot \frac{\mathrm{GLU}}{K_{m\mathrm{GLU}}+\mathrm{GLU}}$

Table S3: Flux equations for the single-cell model (continued).

#	Flux equation
25	$v(\text{GLNT}) = v_{\text{max GLNT}} \cdot \frac{\text{EGLN}}{K_{m\text{EGLN}}+\text{EGLN}}$
26	$v(\text{ASPT}) = v_{\text{max ASPT}} \cdot \frac{\text{EASP}}{K_{m\text{EASP}}+\text{EASP}}$
27	$v(\text{LACT}) = v_{\text{maxf LACT}} \cdot \frac{\text{LAC}}{K_{m\text{LAC}}+\text{LAC}} - v_{\text{maxr LACT}} \cdot \frac{\text{ELAC}}{K_{m\text{ELAC}}+\text{ELAC}}$
28	$v(\text{O2T}) = v_{\text{max O2T}} \cdot \frac{\text{O}_2^{aq}}{K_{m\text{O2}}+\text{O}_2^{aq}}$
29	$v(\text{growth}) = v_{\text{max growth}} \cdot \frac{\text{GLC}}{K_{m\text{GLC}}+\text{GLC}} \cdot \frac{\text{GLN}}{K_{m\text{GLN}}+\text{GLN}} \cdot \frac{\text{ALA}}{K_{m\text{ALA}}+\text{ALA}} \cdot \frac{\text{HIS}}{K_{m\text{HIS}}+\text{HIS}} \cdot \frac{\text{ILE}}{K_{m\text{ILE}}+\text{ILE}} \cdot \frac{\text{LEU}}{K_{m\text{LEU}}+\text{LEU}} \cdot \frac{\text{LYS}}{K_{m\text{LYS}}+\text{LYS}}$ $\cdot \frac{\text{SER}}{K_{m\text{SER}}+\text{SER}} \cdot \frac{\text{THR}}{K_{m\text{THR}}+\text{THR}} \cdot \frac{\text{TYR}}{K_{m\text{TYR}}+\text{TYR}} \cdot \frac{\text{VAL}}{K_{m\text{VAL}}+\text{VAL}} \cdot \frac{\text{MET}}{K_{m\text{MET}}+\text{MET}} \cdot \frac{\text{PHE}}{K_{m\text{PHE}}+\text{PHE}} \cdot \frac{\text{PRO}}{K_{m\text{PRO}}+\text{PRO}}$

Table S4: Estimated mechanistic parameter values for the cell metabolic kinetic model.

Parameter	Value	Parameter	Value
v_{maxHK}	1.75	$K_{i\text{LACtoHK}}$	31.60
$K_{m\text{GLC}}$	1.45	v_{maxfLDH}	3.28
v_{maxrLDH}	0.10	$K_{m\text{PYR}}$	0.21
$K_{m\text{LAC}}$	0.04	$K_{m\text{NADHtoNAD}}$	4.00×10^{-3}
$K_{m\text{NADtoNADH}}$	0.50	v_{maxPDH}	0.22
v_{maxPC}	0.06	v_{maxCS}	0.43
$K_{m\text{AcCoA}}$	0.09	$K_{m\text{OAA}}$	0.08
$v_{\text{maxCITSISOD}}$	1.32	$K_{m\text{CIT}}$	0.39
v_{maxAKGDH}	2.84	$K_{m\text{AKG}}$	2.92
v_{maxSDH}	0.32	$K_{m\text{SUCCOA}}$	0.16
$K_{m\text{FUM}}$	0.16	v_{maxFUM}	0.32
v_{maxMDH}	1.44	$K_{m\text{MAL}}$	0.11
$K_{m\text{NADPtoNADPH}}$	1.00×10^{-3}	v_{maxME}	0.51
v_{maxfGLNS}	0.61	v_{maxrGLNS}	20.28
$K_{m\text{GLN}}$	0.26	$K_{m\text{GLU}}$	0.30
$K_{m\text{NH4}}$	1.71	v_{maxPRO}	2.00×10^{-3}
v_{maxPROr}	2.00×10^{-3}	$K_{m\text{PRO}}$	0.10
$K_{i\text{O2}}$	4×10^{-6}	v_{maxHIS}	0.03
$K_{m\text{HIS}}$	0.08	v_{maxfGLDH}	0.26
v_{maxrGLDH}	0.09	$v_{\text{maxfAlaTA}}$	0.82
$v_{\text{maxrAlaTA}}$	0.10	$K_{m\text{ALA}}$	0.20
v_{maxSAL}	0.01	$K_{m\text{SER}}$	0.01
v_{maxASN}	0.02	$K_{m\text{ASN}}$	0.05
v_{maxfASTA}	3.06	v_{maxrASTA}	0.03
$K_{m\text{ASP}}$	75.79	v_{maxILE}	0.10
$K_{m\text{ILE}}$	0.55	v_{maxLEU}	0.05
$K_{m\text{LEU}}$	0.55	v_{maxTHR}	0.03
$K_{m\text{THR}}$	0.55	v_{maxTRP}	0.10
$K_{m\text{TRP}}$	0.55	v_{maxLYS}	0.05
$K_{m\text{LYS}}$	0.55	v_{maxVAL}	0.05
$K_{m\text{VAL}}$	0.55	v_{maxMET}	0.10
$K_{m\text{MET}}$	0.55	v_{maxPHE}	0.05
$K_{m\text{PHE}}$	0.55	v_{maxTYR}	0.05
$K_{m\text{TYR}}$	0.55	v_{maxresp}	7.00
$K_{m\text{O2}}$	4.00×10^{-6}	$K_{m\text{NADH}}$	4.00×10^{-6}
v_{maxPYRT}	0.17	$K_{m\text{EPYR}}$	0.19
v_{maxAlaT}	0.38	v_{maxGluT}	0.24
v_{maxGlnT}	0.36	v_{maxASPT}	0.40
$K_{m\text{EGLN}}$	1.00	$K_{m\text{EASP}}$	12.82
v_{maxfLACT}	2.97	v_{maxrLACT}	0.60
$K_{m\text{ELAC}}$	0.60	$v_{\text{maxgrowth}}$	0.10

Table S5: Diffusion coefficients for extracellular metabolites at 37°C (10^{-9} m^2/s).

Metabolite	D_i^a	Metabolite	D_i^a
Pyruvate	1.12	Glucose	0.60
Alanine	0.91	Glutamine	0.76
Aspartate	0.741	Glutamate	0.708
Glycine	1.04	Lactate	1.033
Serine	0.891	Ammonia	1.86
O_2	1.163	Arginine	0.327
Asparagine	0.83	Histidine	0.73
Isoleucine	0.641	Leucine	0.73
Lysine	0.626	Tyrosine	0.30
Valine	0.83		

Table S6: Evaluation of metabolites for inclusion in the single cell model for species that are significantly labeled by [U-$^{13}C_3$] pyruvate or exhibit differential labeling between cultures with and without elevated lactate.

Metabolite	Significant Accumulation	Different between High/Low Lac?	Considered Accumulation in Model?	Notes
3PG	Yes	No	No	Low peak intensity
ACO	Yes	No	No	Low peak intensity
AKG	Yes	No	Yes	
ALA	Yes	Yes	Yes	
ASN	Yes	No	Yes	
ASP	Yes	No	Yes	
CIT	Yes	No	Yes	
CYS	No	Yes	No	No concentration measurement available
FUM	No	Yes	Yes	
GLC	Yes	No	No	Low peak intensity
GLN	No	No	No	
GLU	No	No	No	
GLY	No	No	No	
HIS	Yes	No	No	Essential amino acid; low peak intensity
ILE	No	No	No	Essential amino acid
LAC	Yes	Yes	Yes	
LYS	No	No	No	Essential amino acid
MAL	No	No	Yes	
MET	No	No	No	Essential amino acid
PEP	Yes	No	No	Low peak intensity
PHE	No	No	No	Essential amino acid
PRO	No	No	No	
PYR	Yes	Yes	Yes	
SER	No	No	No	
SUC	No	Yes	Yes	
THR	No	No	No	Essential amino acid
TRP	Yes	Yes	No	Essential amino acid
TYR	No	No	No	
VAL	No	No	No	Essential amino acid
